# Targeting Metabolic Dysregulation in Obesity and Metabolic Syndrome: The Emerging Role of N-Acetylcysteine

**DOI:** 10.3390/metabo15100645

**Published:** 2025-09-26

**Authors:** Dorota Magdalena Radomska-Leśniewska, Justyna Niderla-Bielińska, Marek Kujawa, Ewa Jankowska-Steifer

**Affiliations:** 1 Department of Histology and Embryology, Faculty of Medicine, Medical University of Warsaw, Chalubinskiego 5 Str, 02-004 Warsaw, Poland; dorota.radomska-lesniewska@wum.edu.pl (D.M.R.-L.); jniderla@wum.edu.pl (J.N.-B.); 2 Department of Histology and Embryology, Faculty of Medicine, Lazarski University, 02-662 Warsaw, Poland; marek.kujawa@lazarski.edu.pl

**Keywords:** NAC, metabolic modulator, cardiovascular disease, oxidative stress, metaflammation, type 2 diabetes, visceral fat, dyslipidemia, insulin resistance, atherosclerosis

## Abstract

Obesity and metabolic syndrome (MetS), growing global health concerns, are closely linked to the development of insulin resistance, type 2 diabetes, steatotic liver disease, and cardiovascular diseases (CVDs). An increase in visceral adipose tissue, the main symptom of MetS, contributes to systemic metabolic dysfunction, resulting in disturbances in glucose and lipid metabolism, mitochondrial dysfunction, and redox imbalance, which creates a vicious cycle of inflammation and oxidative stress, accelerating comorbidities. N-acetylcysteine (NAC), a precursor to glutathione, with antioxidant and anti-inflammatory properties, is described as a potent metabolic modulator that restores metabolic homeostasis. NAC’s ability to modulate oxidative stress and inflammation may be particularly valuable in preventing or mitigating cardiovascular complications of MetS. The aim of this narrative review is to summarize current evidence from cellular, animal, and human studies on NAC’s impact on metabolic health. MetS affects nearly one-third of the global population; therefore, there is a pressing need for accessible therapeutic strategies. NAC appears to offer potential benefits as an adjunctive agent for individuals with metabolic disturbances, but further research is needed to confirm its efficacy and establish its role in clinical practice.

## 1. Introduction

Overweight and obesity are currently among the world’s major health problems. According to the World Health Organization (WHO) European Regional Obesity Report 2022, nearly two-thirds of adults and one in three children in the WHO European Region are overweight or obese, and unfortunately, this trend is increasing compared to previous years. Given this upward trend, it is estimated that by 2050, nearly two in three adults aged 25 and older will be overweight or obese [1]. The consequence of obesity is an increase in visceral adipose tissue, which, due to its specific metabolic activity, has a negative impact on the functioning of the body. Visceral adipose tissue accumulation is the most common and key component of metabolic syndrome (MetS), which is characterized by the coexistence of abdominal obesity, hyperglycemia, dyslipidemia, insulin resistance, and hypertension [2]. In clinical practice, MetS is typically diagnosed using criteria such as waist circumference (≥102 cm in men, ≥88 cm in women), elevated triglycerides (≥150 mg/dL), reduced HDL cholesterol, high blood pressure, and fasting glucose ≥5.6 mmol/L. To better assess visceral adiposity and cardiometabolic risk, composite indices like the Lipid Accumulation Product (LAP) and Cardiometabolic Index (CMI) have been proposed, with cut-off values of LAP ≥ 49.87 or CMI ≥ 0.56 for women and LAP ≥ 52.76 or CMI ≥ 0.70 for men. These markers show superior diagnostic accuracy compared to traditional measures such as body mass index (BMI) or waist circumference alone [3]. Visceral obesity itself is an independent component of the MetS, and the severity of this obesity is directly related to the prognosis of this condition [4,5]. Obesity predisposes to the development of insulin resistance, type 2 diabetes (T2DM), non-alcoholic fatty liver disease (now known as metabolic dysfunction-associated steatotic liver disease—MASLD), and cardiovascular diseases (CVDs) [6]. CVDs are one of the leading causes of death in the world, and among the most important risk factors for these diseases are metabolic disorders resulting from abdominal obesity, which may lead to the development of hypertension, insulin resistance, and atherogenic dyslipidemia. The pathophysiological mechanisms linking abdominal obesity with CVD are complex and multifaceted [7,8].

Given the complexity of metabolic disturbances in the pathogenesis of CVD, restoring normal metabolism seems to be a viable strategy for improving heart function or preventing further damage. Compounds capable of influencing key metabolic pathways—such as glucose and lipid metabolism, mitochondrial function, or redox homeostasis—in a way that helps restore metabolic balance are referred to as metabolic modulators [9]. To date, metabolic modulators in CVD have been used to modify cardiac energy metabolism, particularly in heart failure (HF) [10], and modulate glucose and lipid transporters, mitochondrial function, and inflammatory signaling [11]. Metabolic modulators encompass a wide range of compounds, including peroxisome proliferator-activated receptor (PPAR) agonists, fibroblast growth factor 21 (FGF21) analogues, thyroid hormone receptor β agonists, and glucagon-like peptide-1 (GLP-1) receptor agonists [12,13,14,15]. Currently, one of the most widely studied and promising metabolic modulators is N-acetylcysteine (NAC) due to its broad spectrum of biological activity relevant to cardiometabolic health. According to numerous studies, NAC may improve insulin sensitivity, reduce oxidative stress, and support the proper function of adipose tissue, which may be beneficial for MetS patients [16]. Therefore, the aim of this paper is to present current information regarding NAC’s impact on MetS symptoms in cell, animal, and human studies. Since MetS and obesity affect more and more patients, it is extremely important to develop new therapeutic strategies not only to effectively manage MetS comorbidities but also to prevent metabolic deregulation. A healthy lifestyle combined with metabolic modulators, such as NAC, may be a future option for high-risk patients, although more clinical trials are needed to evaluate the efficacy of this substance.

## 2. Methodology

This narrative review was conducted to synthesize current knowledge on the role of NAC in MetS, obesity-related metabolic disturbances, and cardiovascular health. We searched the PubMed database using keywords including “NAC”, “metabolic syndrome”, “obesity”, “cardiovascular health”, “insulin sensitivity”, “oxidative stress”, “visceral adipose tissue”, and “leptin signaling”. Particular attention was given to studies published within the last five years, including original research articles, reviews, and experimental studies in both animal and human models. The selection of the literature was guided by relevance to the topic, with emphasis on studies exploring NAC’s biochemical actions, its influence on adipose tissue function, and its potential therapeutic role in mitigating cardiometabolic risk. Additionally, older foundational studies were included when they provided essential background information or mechanistic insights relevant to the topic. The review is structured to first present the pathophysiological mechanisms underlying metabolic disturbances in obesity and MetS, followed by a discussion of NAC’s effects on cellular metabolism and cardiovascular outcomes. Images were created by the authors with the use of image elements provided by Servier Medical Art (https://smart.servier.com/) accessed on 12 August 2025, licensed under CC BY 4.0 (https://creativecommons.org/licenses/by/4.0/).

## 3. MetS and Metabolic Disruption

### 3.1. Visceral Adipose Tissue

There are two types of white adipose tissue, based on its anatomical location: subcutaneous adipose tissue (SAT) and visceral adipose tissue (VAT). SAT is located under the skin, primarily in the abdominal region, buttocks, thighs, and upper limbs. VAT, also known as ectopic fat, accumulates deep in the abdominal cavity, surrounding vital organs such as the liver, intestines, heart (pericardium, epicardium), and skeletal muscle. It can also occur in areas of the body not typically associated with fat storage and containing only small amounts of fat, such as the renal sinus, pancreas, thoracic cage, periaortic sinus, and perivascular areas [8,17]. VAT and SAT differ in their cellular and molecular characteristics, metabolic functions, clinical implications, and prognostic significance [18]. Compared with SAT, VAT consists of larger adipocytes due to enlarged lipid droplets. It is better vascularized and innervated [19]. Adipocyte hypertrophy in VAT is highly correlated with the accumulation of proinflammatory adipokines and immune cells [20]. All types of VAT are metabolically active, insulin-resistant due to a low number of insulin receptors, highly lipolytic, and resistant to the antilipolytic effects of insulin. Compared with SAT, VAT responds better to adrenergic stimulation and has a greater capacity for free fatty acid (FFA) release and glucose uptake. Its metabolic profile contributes to systemic insulin resistance and increases the risk of T2DM, obstructive sleep apnea, and CVD [8,19]. It should be remembered that adipose tissue also accumulates around the heart as epicardial adipose tissue (EAT), which occurs between the outer wall of the myocardium and the visceral layer of the pericardium. Under physiological conditions, EAT provides the heart with mechanical, thermogenic, and metabolic support and releases cytokines and chemokines into blood vessels [21]. In obesity, EAT becomes dysfunctional and contributes to myocardial connective tissue hypertrophy and fibrosis, and through the secretion of proinflammatory factors, reduces the synthesis of adiponectin, an important regulator of glucose and lipid metabolism [8].

### 3.2. Lipid Metabolism and Its Deregulation

Dietary fats are absorbed by enterocytes in the form of FFA and monoglycerides, and then converted into triglycerides (TGs), which, together with cholesterol, phospholipids, and apolipoproteins (primarily ApoB-48), form lipoproteins called chylomicrons, which are distributed through the lymph and blood. TGs in chylomicrons are broken down by lipoprotein lipase into FFA and glycerol, enabling their uptake by target cells, such as adipocytes and muscle cells. Free fatty acids are used for energy production through beta-oxidation in mitochondria [22,23]. Fatty acids in the form of TG are stored in adipocytes, and in the event of glucose deficiency, they are converted into ketone bodies in the liver. The degraded chylomicron remnants are taken up by the liver. Cholesterol is also absorbed and synthesized in the intestines and transported in lipoproteins of varying density [24,25]. Cholesterol is a structural component of cell membranes and is used to produce hormones, vitamin D, or stored as cholesterol esters [26]. A consequence of obesity and VAT accumulation is atherogenic dyslipidemia—a lipid disorder characterized by abnormal levels of cholesterol and TG in the blood. Triglyceride levels increase, while high-density lipoprotein (HDL) cholesterol (which reduces the risk of CVD) decreases. The lipid-overloaded liver produces more small low-density lipoprotein (LDL) particles (key transmitter of cholesterol to the vascular artery wall), which are directly associated with the risk of atherosclerotic cardiovascular events [27,28,29]. Preliminary estimates indicate that approximately 60–70% of obese individuals and 50–60% of overweight individuals suffer from dyslipidemia, a significant risk factor for CVD [30,31].

### 3.3. Glucose Metabolism and Its Deregulation

Dietary carbohydrates, digested into simple, soluble sugars, are transported through enterocytes in the intestinal wall into the circulatory system and then to various tissues [32]. Glucose uptake in skeletal muscle and adipose tissue is regulated by insulin, which translocates the GLUT4 glucose transporter to the cell membrane [33]. Glucose catabolism, leading to ATP production, involves three sequential processes: glycolysis, the tricarboxylic acid cycle (Krebs cycle), and finally oxidative phosphorylation [34]. When excess glucose is available, insulin activates glycogen synthase, initiating glycogenesis in muscle and liver [35]. High blood glucose levels often coexist with insulin resistance—a condition in which cells (especially muscle and adipocytes) respond poorly to insulin. The body compensates by increasing insulin secretion from the pancreas, leading to hyperinsulinemia. Chronic hyperglycemia leads to cell dysfunction and apoptosis, resulting in T2DM [36]. People with T2DM have a higher risk of developing and dying from CVD (such as myocardial infarction, stroke, and heart failure) [37].

### 3.4. Adipokine Profile of Visceral Adiposity

VAT, through its paracrine activity and the secretion of bioactive molecules, i.e., adipokines, participates in the regulation of metabolic, inflammatory, and cardiovascular processes [38]. Obesity disrupts their production, which contributes to the development of many diseases, including CVD [39]. Pro-inflammatory adipokines, including leptin, resistin, visfatin, and chemerin, contribute to the development and progression of chronic inflammation. In contrast, adipokines such as adiponectin, vaspin, apelin, omentin, and isthmin-1 exert anti-inflammatory effects and are associated with improved metabolic homeostasis [40]. Adiponectin is the main cardioprotective adipokine and exerts a protective effect against the development of obesity-related CVD. Its high levels correlate with a lower risk of atherosclerosis, hypertension, and coronary artery disease, while in obesity, its deficiency contributes to the development of chronic inflammation and vascular dysfunction. It has been documented that circulating adiponectin levels are reduced in CVD [41]. Obesity increases VAT volume, which is the main source of leptin, which typically plays a key role in regulating appetite, energy homeostasis, and body weight. However, despite high leptin levels, many obese individuals experience leptin resistance, and high circulating leptin levels can lead to endothelial dysfunction, which is a precursor and major contributor to hypertension, atherosclerosis, and coronary artery disease [8,42].

### 3.5. Integrated Metabolic Disruption in CVD

Metabolism is a multifactorial phenomenon in which individual components are interrelated and exert reciprocal effects on one another, creating, in pathological conditions, a self-perpetuating cycle. Dyslipidemia and hyperglycemia are closely interconnected metabolic disturbances commonly observed in obesity, and together they significantly contribute to the development of CVD [43]. Elevated levels of FFA in obesity promote insulin resistance, which impairs glucose uptake and leads to chronic hyperglycemia [44]. In turn, hyperglycemia exacerbates oxidative stress and inflammatory signaling, further disrupting lipid metabolism and promoting atherogenic lipid profiles. Insulin resistance causes increased lipolysis of TG from adipose tissue and increased glucose production in the liver [22]. Moreover, hyperglycemia can modify lipoproteins through non-enzymatic glycation, making them more prone to oxidation and less efficiently cleared from circulation. These modified lipoproteins contribute to endothelial dysfunction and accelerate atherosclerotic plaque formation, a risk factor of atherosclerotic CVD [45,46]. Hyperglycemia, lipid accumulation in tissues, chronic low-grade inflammation, elevated leptin levels, endothelial dysfunction, and impaired mitochondrial function can lead to increased production of reactive oxygen species (ROS) [47]. ROS are integral components of many cellular pathways. Under physiological conditions, in small amounts, ROS exhibit antimicrobial and signaling properties and are neutralized by antioxidant systems containing glutathione (GSH) [48,49]. However, in the course of many disorders, including obesity, their excessive production occurs, leading to oxidative stress. The main sources of cellular ROS are mitochondria and NADPH oxidases [50]. It has been shown that in endothelial cells (ECs) and vascular myocytes, increased levels of glucose and FFA can stimulate ROS production via phosphokinase C (PKC)-dependent activation of NAD(P)H oxidase [51].

The coexistence of dyslipidemia, hyperglycemia, and oxidative stress creates a pro-inflammatory environment that plays a key role in the development of CVD [52]. Progressive hypertrophy of VAT leads to adipocyte stress and local hypoxia, which activates the hypoxia inducible factor 1 (HIF-1) gene. This predisposes to adipocyte necrosis and macrophage infiltration [53]. M1 macrophages secrete pro-inflammatory cytokines (e.g., interleukins (IL-1β and IL-6), monocyte chemoattractant protein-1 (MCP-1), and tumor necrosis factor α (TNF-α)), contributing to local and systemic inflammation and insulin resistance [54].

Lipoproteins are now increasingly recognized as important modulators of vascular inflammation and atherosclerosis. HDL not only removes accumulated cholesterol and phospholipids but also has potent anti-inflammatory effects, while triglyceride-rich lipoproteins such as LDL can stimulate inflammation and foam cell formation [55]. The expansion of adipose tissue is accompanied by an increased release of adipokines into circulation, encompassing both pro-inflammatory and anti-inflammatory mediators. Leptin, a pro-inflammatory adipokine, can directly stimulate the production of various inflammatory mediators—including cytokines such as IL-6, IL-12, IL-18, and TNFα; chemokines like IL-8 and MCP-1; and lipid-derived molecules such as prostaglandin E2 (PGE2), cysteinyl leukotrienes (cysLT), and leukotriene B4 (LTB4) [56,57].

### 3.6. Mitochondrial Dysfunction in Obesity, MetS, and CVD

Dyslipidemia, hyperglycemia, and insulin resistance disrupt normal mitochondrial function, and their dysfunction becomes a central element in the pathogenesis of MetS and its cardiovascular complications. In the healthy heart, more than 95% of ATP is produced by oxidative phosphorylation in mitochondria. The remaining energy comes mainly from glycolysis, with a small contribution from direct ATP generation in the Krebs cycle [58]. In CVD, mitochondrial dysfunction is characterized by impaired oxidative phosphorylation, excessive production of ROS, impaired mitochondrial dynamics, and impaired calcium metabolism. These changes contribute to endothelial dysfunction, myocardial remodeling, and progression of HF [59].

At the molecular level, mitochondrial dysfunction can be considered in relation to several signaling pathways. Elevated ROS levels activate the nuclear factor kappa-light-chain enhancer of activated B cells (NF-κB), which promotes chronic inflammation and contributes to atherosclerosis and cardiac remodeling [60].

Cellular stress leads to activation of the mitogen-activated protein kinase (MAPK) pathway, which, under oxidative conditions, drives cardiomyocyte hypertrophy and apoptosis [61]. Inhibition of the phosphoinositide 3-kinase/protein kinase B (PI3K/AKT) pathway during oxidative stress increases cellular damage and disrupts mitochondrial integrity [62]. Dysfunction of AMP-activated protein kinase (AMPK), a key regulator of energy homeostasis, impairs lipid and glucose metabolism and reduces mitochondrial biogenesis [63]. Reduced activity of sirtuin 3 (SIRT3), a mitochondrial deacetylase that normally maintains redox balance and regulates respiratory enzymes, is associated with increased oxidative stress and impaired mitochondrial function [64,65].

**Figure 1:**
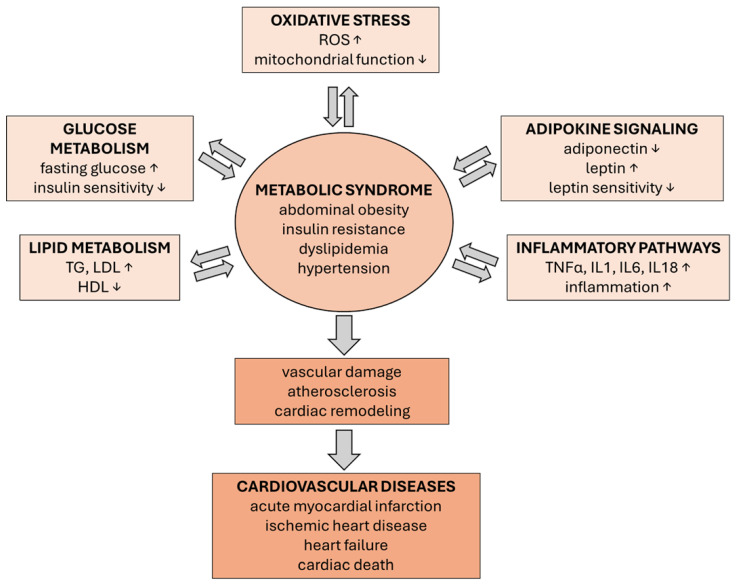
Metabolic disruption in MetS and its comorbidities.

Metabolic disruption in MetS is summarized in Figure 1.

## 4. N-Acetylcysteine

NAC, an acetylated form of the amino acid L-cysteine, has been used clinically since the 1960s as a mucolytic agent and since the 1980s as an effective antidote for acetaminophen overdose. Its therapeutic efficacy is primarily attributed to its antioxidant and anti-inflammatory properties, which help restore cellular redox balance and protect against damage caused by ROS. In addition to these effects, NAC exhibits cytoprotective, antibacterial, and antimicrobial effects, making it a versatile treatment option for various human diseases [66]. These include metabolic disorders such as diabetes and obesity, cancer, inflammatory bowel disease, neurological conditions, hypertension, as well as pulmonary diseases, autoimmune diseases, and CVDs [67]. Oxidative stress and inflammation are key drivers in the pathogenesis and progression of these disorders. NAC’s ability to modulate these processes has positioned it as a promising therapeutic agent in conditions characterized by disrupted redox homeostasis [16].

NAC exerts its biological effects through several mechanisms. Most importantly, NAC serves as a precursor for the intracellular synthesis of GSH, the body’s most potent endogenous antioxidant [66]. After administration, NAC is metabolized into L-cysteine, which increases intracellular cysteine levels and thereby enhances the rate of GSH synthesis. GSH levels are often reduced during oxidative stress and chronic inflammation, conditions commonly seen in aging and disease. By restoring GSH levels, NAC helps rebalance the cellular redox state. This enables NAC to influence redox-sensitive signaling pathways and transcriptional activity, which are crucial for regulating inflammation, cell survival, and stress responses [68].

The strong antioxidant properties of NAC are also related to its structure. It is a sulfhydryl substance, possessing free SH groups, thanks to which it directly neutralizes ROS (namely hydroxyl radicals (•OH)) and hypochlorous acid (HOCl), but does not react directly with hydrogen peroxide (H_2_O_2_) or superoxide anions (O_2_•−) [69]. NAC’s chemical structure allows it to act as a nucleophile, meaning it can donate electrons to other molecules. This property enables NAC to bind to and neutralize electrophilic toxins, heavy metals, and ROS. By donating electrons, NAC helps to detoxify these substances, preventing them from damaging cells and tissues. This electron-donating ability is a key part of NAC’s antioxidant and protective role in the body [70]. NAC also exerts indirect antioxidant action through conversion into hydrogen sulfide and sulfane sulfur species, which expose antioxidant and cytoprotective properties [71]. NAC also influences the activity of enzymes like superoxide dismutase (SOD) and catalase, which transform ROS into less harmful substances. It should also be noted that the effect of NAC can sometimes be the opposite, i.e., it can have pro-oxidant properties as a result of the autoxidation process, causing formation of H_2_O_2_ in the presence of O_2_ [72]. NAC is primarily an antioxidant; however, it may, rarely, exhibit pro-oxidant activity under certain conditions. First and foremost, high NAC concentrations can disrupt the redox balance and promote the formation of ROS. Furthermore, the presence of transition metals such as iron or copper can trigger reactions, generating harmful radicals. Also, impaired GSH synthesis can lead to NAC accumulation and oxidative stress. Pathological conditions, such as chronic inflammation or cancer, have also been reported to alter the redox properties of NAC [73].

By restoring redox balance, NAC influences redox-sensitive transcription factors and pathways that regulate, among others, inflammation, cell survival, and apoptosis. It is known to inhibit the activation of stress-responsive kinases such as c-Jun N-terminal kinase (JNK) and MAPK, while promoting extracellular signal-regulated kinase (ERK) signaling, which supports neuroprotection and cell survival [74]. NAC also activates the PI3K/AKT pathway, which plays a central role in preventing oxidative stress-induced hypertrophy and apoptosis in cardiomyocytes [75,76]. Furthermore, NAC suppresses pro-inflammatory signaling cascades, including NFκB, activator protein 1 (AP-1), stress-activated protein kinases/cyclin-dependent kinase inhibitor (SAPK/INK), c-Fos, JNK, and signal transducers and activators of transcription (STATs), leading to reduced expression of cytokines such as IL-6, IL-8, and TNF-α [77,78,79,80]. In addition, NAC protects mitochondrial and genomic integrity by reducing oxidative damage and regulating apoptotic enzymes, including caspases 3, 8, and 9, particularly in cardiac and neuronal cells [81].

NAC has demonstrated protective effects on DNA damage [70]. It reduces oxidative DNA damage, as NAC administration lowers the levels of 8-hydroxyguanine (8-OH-G), a key biomarker of ROS-induced genotoxicity. NAC also influences gene expression through epigenetic mechanisms, including modulation of DNA methylation [82], and by altering the activity of histone-modifying enzymes such as histone deacetylases (HDACs) and histone acetyltransferases (HATs), which affect chromatin structure and gene accessibility [83]. Additionally, NAC supports the expression of non-coding RNAs, including microRNAs (e.g., miR-141, miR-3, miR-13) and long non-coding RNAs (e.g., lncRNA-EN-181), which are involved in regulating cellular responses to oxidative stress [84,85]. These combined actions contribute to NAC’s role in protecting cells from oxidative damage and maintaining genomic stability.

## 5. NAC and MetS—Evidence from Cellular, Animal, and Human Studies

Standard therapeutic management for patients with obesity, MetS, and CVD includes lifestyle changes (weight reduction, diet, physical activity), pharmacotherapy (statins, angiotensin converting enzyme (ACE) inhibitors, beta-blockers, antidiabetic medications), and interventional treatment for acute myocardial infarction (e.g., coronary angioplasty). Lately, there has also been growing interest in metabolic modulators—compounds that can help to restore metabolic balance [86]. Growing evidence from both cell culture, animal, and human studies suggests that NAC may offer therapeutic benefits in MetS-associated comorbidities, including CVD, by improving redox balance, glucose metabolism, insulin sensitivity, lipid metabolism, and inflammation, although some data seem contradictory, and a more in-depth analysis is surely necessary to fully understand NAC’s actions (summarized in Table 1). Additionally, NAC is often combined with other compounds, e.g., glycine, apocynin, betaine, L-carnitine, methionine, or pyridoxine [87,88,89,90]. In vitro and in vivo experiments show that NAC can inhibit lipid accumulation by targeting adipogenic transcription factors such as peroxisome proliferator-activated receptor gamma (PPARγ) and CCAAT/enhancer binding protein beta (C/EBPβ) and improve insulin sensitivity through augmenting the PI3K/AKT pathway (summarized in Table 2 and reviewed by [91]).

### 5.1. Effect of NAC on Glucose Metabolism and Its Deregulation

A lot of studies based on in vitro and animal models have shown that NAC may exert significant effects on glucose metabolism and insulin signaling. In Olson et al.’s study [98], NAC was used to investigate its ability to reverse insulin resistance in 3T3-L1 adipocytes with silenced peroxiredoxin-3 (Prdx3), a mitochondrial antioxidant enzyme, which mimics oxidative stress conditions [98]. Treatment with NAC restored insulin sensitivity and stimulated glucose uptake via a reduction in mechanistic target of rapamycin complex 2 (mTORC2) oxidation and upregulation of AKT phosphorylation. Moreover, NAC seemed to protect mitochondrial function without triggering endoplasmic reticulum (ER) stress or mitochondrial unfolded protein response (mtUPR). Similar results were observed in skeletal muscle fibers isolated from high-fat-diet (HFD) mice by Russell-Guzman et al. [116]. The treatment with NAC significantly reduced oxidative stress markers, including lipid peroxidation and peroxiredoxin 2 dimerization, and inhibited activation of the ROS/TXNIP/NLRP3 inflammasome pathway. Moreover, NAC reduced caspase-1 activity and restored insulin-dependent glucose uptake and AKT phosphorylation, but at the same time impaired insulin signaling in control (non-insulin-resistant) muscle fibers, suggesting its effects are context-dependent and influenced by the redox state [116]. In Alnahdi et al.’s investigation [107], NAC was used to mitigate glucolipotoxicity-induced damage in pancreatic β-cells. Cells exposed to high glucose and palmitic acid showed increased apoptosis, mitochondrial dysfunction, and oxidative stress. NAC treatment significantly reduced cell death, preserved mitochondrial membrane potential and ATP production, and restored redox balance. It also reversed suppression of autophagy by increasing autophagy-related protein light chain 3 (LC3-II) expression and modulating mTOR/AMPK signaling [107].

Animal studies also show the beneficial impact of NAC on insulin sensitivity in insulin-responsive tissues, mainly via restoration of proper levels of phosphorylated AKT. In Argaev-Frenkel et al.’s study [117], in diabetic KK-Ay mice, NAC showed tissue-specific, dose-dependent effects on glucose metabolism and insulin signaling. It preserved isolated pancreatic islet function but induced reductive stress in insulin-responsive tissues, impairing insulin signaling and glucose uptake via reduced AKT phosphorylation. In vivo, NAC improved glucose tolerance in diabetic mice but disrupted insulin signaling in healthy C57BL/6 mice, indicating its effects depend on tissue oxidative status [117]. Schuurman et al. [118] showed that NAC improves glucose metabolism and β-cell function when administered before and during HFD-induced diabetes in mice. Long-term treatment was essential, as short-term (10 weeks) NAC had no effect. Benefits were linked to its antioxidant and anti-inflammatory actions, including GSH precursor activity and NF-κB inhibition [118]. NAC, administered to obese Swiss mice, significantly reduced fasting blood glucose and improved insulin sensitivity via restoration of phosphorylated AKT levels, lowered nitrite and protein carbonyl levels, indicating reduced oxidative damage, and increased glutathione peroxidase (GPx) protein levels, enhancing antioxidant defense [100]. Similar results were reported by Pieri et al. [97], who showed that NAC, administered orally, significantly improved insulin sensitivity and reduced fasting glucose levels without affecting body weight, food intake, or adiposity. The treatment led to a reduction in both oxidative stress and inflammation markers linked to obesity. Importantly, NAC increased phosphorylation of insulin receptor substrate 1 (IRS1) and AKT, and enhanced IRS1/PI3K association, suggesting improved insulin signaling [97]. Kaneto et al. [119] demonstrated, in a study performed on diabetic db/db (leptin receptor-deficient) mice, that intravenous perfusion of NAC during a hyperglycemic clamp improves insulin sensitivity and increases peripheral glucose uptake as well as upregulation of pancreatic-duodenal homeobox-1 (PDX-1) amounts, responsible for transcription of the insulin gene [119]. In the study by Straub et al. [87], NAC was used in combination with apocynin (a potent antioxidant) to investigate its effects on glucose metabolism and insulin resistance in inducible fat-specific insulin receptor knockout (iFIRKO) mice. Treatment with an antioxidant cocktail delayed the onset of hyperglycemia and reduced its severity, lowering blood glucose and improving insulin sensitivity, but also reducing food intake and water consumption, suggesting a systemic metabolic impact. In leptin-deficient ob/ob mice, antioxidant supplementation rapidly reduced hyperglycemia and normalized hyperphagia, showing antioxidant therapy as a successful tool to improve glucose homeostasis in both acute and chronic models of diabetes and obesity [87]. Similar effects, although tissue-specific, were shown in insulin resistance induced by prolonged elevation of plasma FFA (Intralipid plus heparin infusion) in Wistar rats. While NAC administration did not prevent hepatic insulin resistance caused by FFA, it effectively prevented peripheral insulin resistance, since skeletal muscle showed increased oxidative stress markers after FFA elevation, including protein carbonyls, which NAC successfully suppressed [120]. Markers of oxidative stress in the liver (protein carbonyls, malondialdehyde (MDA), and reduced glutathione/oxidised glutathione (GSH/GSSG) ratio) were not elevated by FFA, suggesting oxidative stress was not the primary driver of hepatic insulin resistance in this model [120].

It is worth mentioning that antioxidant therapy, including NAC, may interfere with glucose meter readings. A study by Grzych et al. [121] discussed the clinical implications for patients undergoing antioxidant therapy, particularly those with MetS. NAC was shown to cause falsely elevated glucose readings on several commercial glucose meters, especially at mid and high plasma concentrations, which could lead to inappropriate administration of glucose-lowering drugs [121].

### 5.2. From Oxidative Stress and Inflammation to Senescence and Aging

The interplay between MetS, CVD, and aging is increasingly attributed to chronic inflammation and oxidative stress, which promote cellular senescence and tissue dysfunction [122,123]. Visceral obesity, a hallmark of MetS, contributes to systemic metabolic derangement by inducing oxidative stress and elevating proinflammatory cytokines in adipose tissue, leading to the senescence-associated secretory phenotype (SASP) [122,124,125]. Senescent cells accumulate in metabolically active tissues, including the heart, where they exacerbate fibrosis, hypertrophy, and mitochondrial dysfunction—features shared by both obesity and aging [123]. These overlapping mechanisms support the concept of obesity as a driver of premature aging and cardiovascular decline.

In VAT from obese patients (BMI > 40), NAC reduced SA-β-gal activity and expression of p16, p21, and IL-6, indicating anti-senescent and anti-inflammatory effects. Samples were collected during bariatric surgery and treated ex vivo [126]. In a similar study of 40 obese individuals (BMI > 35), 4-week NAC supplementation (600 mg/day) reduced SA-β-gal activity and expression of p16 and IL-6 in VAT, but had no effect on lipid profiles or anthropometric measures [127].

In a pilot study with eight older adults (71–80 years), 24-week GlyNAC supplementation (glycine + NAC) corrected GSH deficiency, reduced oxidative stress (e.g., TBARS), improved mitochondrial function, lowered pro-inflammatory markers (IL-6, TNF-α, CRP), and enhanced endothelial function, genomic stability, cognitive performance, and physical capacity (gait speed, grip strength, and exercise tolerance). Fat mass and waist circumference decreased, though muscle mass remained unchanged. Most benefits declined after 12 weeks of discontinuation, indicating the need for ongoing supplementation [88]. A randomized placebo-controlled clinical trial confirmed GlyNAC’s ability to reverse multiple aging-related defects in older adults. In addition to restoring GSH levels and reducing oxidative stress and inflammation, GlyNAC significantly lowered insulin resistance and fasting insulin. NAC contributed to improved nutrient sensing and increased GLUT4 expression. Genomic stability was enhanced, as shown by reduced levels of 8-OH-G and Phospho-H2A Histone Family Member X (pH2AX), markers of DNA damage [128].

### 5.3. Effect of NAC on Adipogenesis, Visceral Obesity, Lipid Metabolism, and Its Deregulation

Obesity is characterized by excessive TG accumulation in VAT and SAT, leading to enlarged adipocytes, inflammation, and altered adipokine secretion. It seems that NAC may influence adipocyte differentiation and lipid metabolism through redox-sensitive transcriptional regulation. NAC was shown to inhibit adipocyte differentiation in 3T3-L1 cells, which are used for adipocyte differentiation studies, by downregulating PPARγ and C/EBPβ, the main adipogenic transcription factors. Moreover, NAC reduced intracellular ROS levels and suppressed the activities of antioxidant enzymes SOD and GPx. It significantly decreased TG accumulation and elevated intracellular GSH content early in the differentiation process, suggesting its antioxidant effect was mediated via GSH. NAC did not interfere with insulin-induced AKT phosphorylation, indicating its action was not through insulin receptor disruption [129]. Additionally, NAC suppressed the expression of key obesity-related proteins, including fatty acid binding protein 4 (FABP4), monoamine oxidase A (MAOA), heat shock protein 70 (HSP70), aminoacylase-1 (ACY-1), and transketolase (TKT) in 3T3-L1 cells [92]. Similar results were obtained by Soto et al. [113], where incubation of 3T3-L1 cells with NAC inhibited the phosphorylation of ERK1/2 and JNK1/2, key kinases involved in proliferation and adipogenesis. Additionally, NAC increased the expression of mitochondrial enzymes citrate synthase and fumarate hydratase, suggesting enhanced aerobic respiration, while decreasing the expression of MAOA, a mitochondrial source of ROS. These molecular changes correlated with reduced TG accumulation and adipogenic marker expression [113]. Consistent findings were observed in studies on mouse embryonic fibroblasts, where NAC significantly decreased lipid content and TG accumulation, and similarly inhibited ERK1/2 and JNK1/2 phosphorylation, supporting its anti-adipogenic effects [130]. This is in line with results obtained by Li et al. [131], where NAC was used as an ROS scavenger to investigate its role in adipocyte differentiation in 3T3-L1 cells. The study found that knockdown of metallothionein 3 (MT3), a protein known for its antioxidant properties, led to elevated ROS levels during early adipogenesis, and treatment with NAC effectively reversed this increase in ROS, suggesting its capacity to mitigate oxidative stress in differentiating adipocytes. This reduction in ROS correlated with a suppression of adipocyte differentiation, indicating that NAC can interfere with the early stages of adipogenesis. The study also showed that NAC treatment restored the expression of ROS scavenging genes such as catalase, Gpx1, Sod1, and Sod2, which were downregulated by MT3 overexpression [131].

Results obtained in animal models also confirm the positive impact of NAC on lipid metabolism and adiposity. In an HFD-induced obese mouse model, multi-ingredient metabolic cofactor (MC) therapy—including NAC—reduced weight gain, improved glucose and insulin tolerance, and lowered fasting glucose, insulin, and HOMA-IR scores. It enhanced lipid oxidation, reduced adipocyte hypertrophy in VAT, and upregulated lipolytic and fatty acid oxidation genes such as adipose triglyceride lipase (Atgl), perilipin 1 (Plin1), and acyl-CoA oxidase 1 (Acox1). In BAT, NAC reduced fat accumulation and increased thermogenic gene expression (Ucp1, Pgc1a, and Dio2). The cocktail also improved lipid profiles, though NAC’s specific contribution remains unclear due to synergistic effects of all components [89].

Metabolic disorders may also affect bone marrow adipose tissue (BMAT), which is considered to be an important source of paracrine factors that may influence bone turnover, protect osteoblasts from lipotoxicity, and maintain bone marrow hematopoietic function and niche integrity [132,133]. In human bone marrow stromal cells differentiated into adipocytes, NAC reduced lipid droplet accumulation and enhanced antioxidant activity, as shown by increased heme oxygenase-1 (HO-1) expression and reduced ROS. NAC upregulated key metabolic and anti-inflammatory markers, including SIRT1, HO-1, adiponectin, and PPARα/δ, while downregulating IL-6. It also reversed the suppression of diacylglycerol acyltransferase 1 (DGAT1) and PPARγ and increased FABP4 expression, indicating improved lipid metabolism and reduced lipotoxicity, supporting NAC’s role in restoring BMAT function [93].

**Figure 2:**
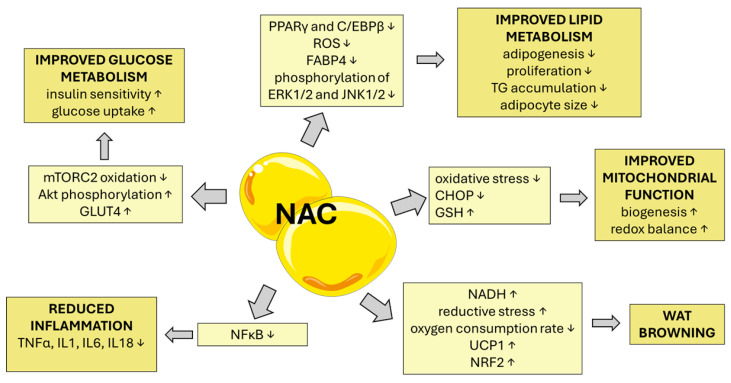
Effect of NAC on adipose tissue—evidence from cellular and animal studies.

NAC’s influence on WAT is summarized in Figure 2.

### 5.4. Effect of NAC on Hepatic Lipid Accumulation

MetS is often associated with MASLD. MASLD, driven by metabolic dysfunction and hepatic fat accumulation, is closely associated with an increased risk of CVD due to shared inflammatory and metabolic pathways [134]. In a rat model of MASLD induced by a high-fat diet, NAC administered at 500 mg/kg/day for eight weeks reduced hepatic levels of lipotoxic sphingolipids, such as ceramide and dihydroceramide, while increasing sphingosine-1-phosphate (S1P) and sphinganine-1-phosphate (SA1P), which are linked to improved insulin sensitivity. NAC modulated key enzymes in sphingolipid metabolism and enhanced insulin signaling via increased phosphorylation of AKT, glycogen synthase kinase-3α/β (GSK3α/β), and P70 S6 kinase. Histological analysis showed reduced lipid accumulation and fibrosis markers, indicating improved liver function and reduced inflammation [110]. A randomized controlled pilot trial was designed to evaluate the effect of MetioNac^®^, which consists of S-adenosyl-L-methionine, NAC, thioctic acid, and vitamin B6, in 15 patients with MetS at risk for MASLD for 3 months. Patients and the control group were also prescribed a semi-personalized Mediterranean diet for weight loss. MetioNac^®^ normalized lipid parameters in the study group, as evidenced by reduced TG and very low-density lipoprotein (VLDL) levels. Both the study and control groups demonstrated a visible weight loss. The authors concluded that MetioNac^®^ may be effective in overweight patients with MetS who suffer from hyperlipidemia and insulin resistance, although the risk of bias was calculated as moderate for this study due to the small sample size, high dropout rate, lack of blinding, and rather exploratory design [90].

### 5.5. Effect of NAC on Mitochondrial Dysfunction in Obesity, MetS, and CVD

A key mechanism of NAC involves its role as a precursor to glutathione, a potent endogenous antioxidant that plays a critical role in neutralizing ROS. NAC supplementation may mitigate oxidative stress and also inflammation, which are among the key drivers of metabolic dysfunction on the cellular level. In cell culture models, NAC consistently reduced ROS levels in adipocytes and preadipocytes exposed to high glucose or palmitic acid [96,107]. NAC was shown to mitigate oxidative stress and reduce C/EBP homologous protein (CHOP) accumulation in adipocytes under metabolic stress. High glucose conditions increase ROS production, activating the nuclear factor erythroid 2-related factor 2 (NRF2) pathway, which plays a crucial role in the protection against oxidative stress and is activated by different stressors. Upregulating HO-1, an antioxidant marker, via NAC treatment decreased intracellular fumarate levels in adipocytes matured in high glucose, which in turn lowered protein succination—a marker of mitochondrial stress. NAC also restored GSH levels and reduced ROS accumulation, contributing to improved redox balance. This antioxidant effect led to decreased CHOP stability and reduced expression of HO-1. Importantly, NAC restored the secretion of IL-13, an anti-inflammatory cytokine suppressed by CHOP accumulation, indicating a reversal of CHOP-mediated transcriptional repression [96]. Similarly, NAC reversed oxidative stress and inflammation in neuregulin 4-deficient adipocytes, restoring insulin receptor and GLUT4 protein levels and inhibiting NFκB activation. Treatment with NAC for 24 hours significantly reduced H_2_O_2_ levels and completely abrogated TNFα gene expression. NAC also increased IκB protein levels, inhibiting NFκB-mediated inflammation, and partially restored insulin receptor and GLUT4 protein content [105].

Animal studies corroborate these findings, although some data are contradictory. NAC supplementation in HFD rats reduced body weight, normalized plasma FFA, and decreased adipocyte hypertrophy and macrophage infiltration in both VAT and SAT, indicating reduced inflammation. NAC decreased expression of fatty acid transporters such as fatty acid translocase (FAT/CD36) and fatty acid binding protein (FABPpm) in VAT and SAT, and also reduced long-chain fatty acid transport protein 1 (FATP1) expression in SAT on both mRNA and protein levels, although these results were inconsistent, probably due to posttranscriptional modifications. NAC also enhanced the expression of β-hydroxyacyl-CoA dehydrogenase (β-HAD), a marker of mitochondrial β-oxidation, especially in VAT, indicating increased fatty acid oxidation [95]. Similarly, prolonged treatment of adipocytes with NAC induced browning via the NRF2/HO-1 signaling axis, marked by elevated expression of uncoupling protein 1 (UCP1), peroxisome proliferator-activated receptor gamma coactivator 1-alpha (PGC1α/β), and mitochondrial biogenesis genes. In vivo, in the HFD mouse model, high-dose NAC reduced fat mass gain, lowered insulin levels, and enhanced mitochondrial oxygen consumption rate in inguinal WAT. These effects were absent in adipocyte-specific NRF2 knockout mice, indicating that NRF2 mediates NAC-induced mitochondrial adaptations [111]. On the contrary, NAC inhibited UCP1 expression and thermogenesis in adipocytes by blocking p38 MAPK activation. NAC, along with other antioxidants like butylated hydroxyanisole (BHA), suppressed both basal and cold- or cAMP-induced UCP1 expression in cultured brown adipocytes and in vivo. This suppression was linked to inhibition of ROS accumulation and subsequent p38 MAPK activation. NAC treatment reduced mitochondrial uncoupling and thermogenic capacity, leading to increased lipid accumulation in brown adipose tissue (BAT). A similar outcome was observed in PG-Sn2 mice (engineered to overexpress sestrin2 in adipose tissue). Sestrin2 is a stress-inducible protein that suppresses ROS accumulation and regulates AMPK/mTORC1 signaling, and loss of this protein leads to insulin resistance, fat accumulation, and mitochondrial dysfunction. Sestrin2 inhibited UCP1 expression, leading to reduced thermogenesis and increased fat accumulation. This suggests that ROS are necessary for proper UCP1 expression and BAT function, and although antioxidants like NAC can reduce oxidative stress, they may inadvertently impair beneficial metabolic processes such as thermogenesis [135].

In catalase-knockout mice fed HFD, which are prone to obesity due to elevated oxidative stress, NAC restored AMPKα phosphorylation and improved mitochondrial function. NAC treatment significantly reduced body weight gain, fat mass, and improved lean mass. It also lowered H_2_O_2_ concentrations in epididymal fat and downregulated nicotinamide adenine dinucleotide phosphate oxidase 4 (NOX4) protein level, which are associated with ROS production. In vitro, NAC reversed H_2_O_2_-induced lipogenesis in 3T3-L1 adipocytes, supporting its role in mitigating oxidative stress-driven adipocyte hypertrophy [108].

As mentioned earlier, NAC may rarely have an oxidative effect instead of an antioxidant effect under certain conditions, especially in high concentrations [73]. Chronic NAC treatment was found to induce reductive stress in adipocytes, increasing intracellular GSH but failing to prevent ROS accumulation after β3-adrenergic stimulation. NAC enhanced mitochondrial ROS production, reduced oxygen consumption, and increased lactate levels, indicating a shift toward glycolytic metabolism. In vivo, in healthy mice, NAC suppressed browning of WAT and reduced mitochondrial activity in BAT, even without adrenergic input. It also upregulated mitochondrial antioxidant proteins, suggesting elevated mitochondrial oxidative stress. These effects were linked to activation of the NRF2 pathway and impaired fatty acid clearance, resulting in increased fat pad size and dysfunctional adipose metabolism. The findings caution against chronic antioxidant use in metabolically healthy individuals [136].

### 5.6. Effects of NAC on Chronic Metabolic Inflammation (Metaflammation) and Immune Modulation

NAC’s anti-inflammatory effects are closely tied to its antioxidant properties. In human macrophages, hypoxia amplified palmitate (PA)-induced inflammation by increasing IL-6 and IL-1β expression, linked to activation of JNK and p38 MAPK pathways. This effect was independent of ER stress. NAC and the mitochondria-targeted antioxidant MitoTEMPO suppressed hypoxia-enhanced cytokine expression and MAPK activation, but had no effect under normoxic conditions. Conditioned medium from hypoxic, palmitate-treated macrophages triggered inflammatory responses in adipocytes, increasing IL-6 and MCP-1, and reducing adiponectin. These effects were mitigated by NAC or JNK inhibition, highlighting NAC’s selective anti-inflammatory role under hypoxia [104]. Similar results were observed in 3T3-L1 adipocytes treated with high glucose. The addition of NAC significantly reduced the elevated levels of ROS and 8-OH-G and normalized the aberrant expression and secretion of pro-inflammatory adipokines—IL-18, MCP-1, and plasminogen activator inhibitor-1 (PAI-1)—and restored adiponectin levels. These effects were observed in both stable and intermittent glucose conditions, with intermittent exposure showing more pronounced dysregulation [101]. In adipocytes with knockdown of the SelenoM gene, NAC partially reversed oxidative stress and inflammatory responses. NAC suppressed the expression of pro-inflammatory cytokines such as TNF-α, IL-6, IL-1β, and inducible nitric oxide synthase (iNOS) in both SelenoM-deficient adipocytes and co-cultured macrophages, by NF-κB deactivation, a key driver of inflammation in macrophages. NAC also improved glucose metabolism by restoring phosphorylation of IRS1, AKT, and GSK3β, and increased GLUT4 expression in SelenoM-knockdown mice. It diminished M1 macrophage polarization and cytokine release, indicating its anti-inflammatory potential [115].

In DNA polymerase η knockout (pol η−/−) mice, NAC was used to investigate its effects on DNA damage-induced adipocyte senescence and metabolic abnormalities. NAC significantly reduced oxidative DNA damage in adipose tissue, as measured by comet assay and markers like H2A histone family member X (γ-H2AX), ataxia-telangiectasia mutated (ATM), p53, and p21. NAC also suppressed NF-κB activation and lowered proinflammatory cytokines IL-6, TNF-α, and MCP-1. Importantly, NAC attenuated adipocyte hypertrophy and senescence markers such as SA–β-gal and p16, and improved insulin sensitivity and glucose tolerance. Interestingly, NAC had minimal impact on wild-type mice [109]. Protective effects of NAC against insulin resistance and adipose tissue inflammation were shown in advanced glycated albumin (AGE-albumin) treated healthy rats. Chronic administration of AGE-albumin to healthy rats led to increased macrophage infiltration in adipose tissue, reduced GLUT4 protein levels, and impaired insulin sensitivity, despite no changes in body weight or plasma lipids. NAC treatment counteracted these effects by reducing lipid peroxidation, decreasing macrophage numbers (without significant changes in M1 (CD11b) or M2 (CD206) markers), and enhancing the expression of GLUT4 and PPAR-α. Although NAC did not fully restore GLUT4 protein levels, it improved glucose tolerance and reduced inflammatory gene expression [94].

NAC significantly reduced arachidonic acid (AA) levels in myocardial phospholipid and TG fractions in male Wistar rats fed an HFD, indicating a suppression of lipid-driven inflammation. It downregulated cyclooxygenase-2 (COX-2) and arachidonate 5-lipoxygenase (5-LOX) expression, leading to decreased levels of proinflammatory eicosanoids such as PGE2, LTB4, and leukotriene C4 (LTC4), while increasing anti-inflammatory lipoxin A4 (LXA4). NAC also inhibited NF-κB activation, resulting in reduced secretion of inflammatory cytokines, including TNF-α and IL-1α. Furthermore, NAC enhanced antioxidant defences by elevating GSH and catalase levels and reduced oxidative damage markers like MDA and 4-hydroxynonenal (4-HNE). These effects were observed both in HFD-fed and standard diet-fed rats [106]. Similar results were obtained in the diabetic-wound-healing rat model. Diabetic rats exhibited elevated ROS levels and increased expression of nod-like receptor protein 3 (NLRP3), caspase-1, apoptosis-associated speck-like proteins (ASCs), and IL-1β, contributing to persistent inflammation and delayed wound closure. NAC effectively reduced ROS production, suppressed NLRP3 inflammasome activation, decreased IL-1β secretion, and improved wound healing, including faster re-epithelialization and reduced healing time [112].

**Figure 3:**
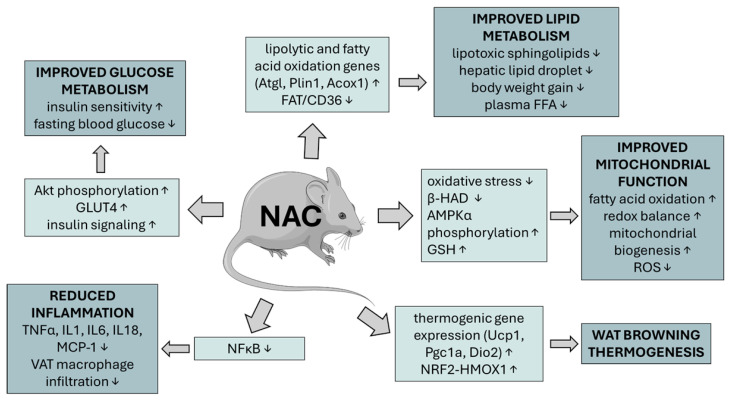
Effect of NAC on obesity and MetS animal models.

NAC’s impact on animal MetS models is summarized in Figure 3.

### 5.7. Obesity and the Brain: A Self-Perpetuating Neuro-Metabolic Disorder

NAC can also exert a positive effect on HFD-induced neurodegeneration by targeting oxidative stress, inflammation, and cell death mechanisms, as well as improve cognition and reduce addiction-like behaviors towards high-fat and high-sugar diets. In Wistar rats fed an HFD and treated with NAC, weight gain, BMI, hyperglycemia, and insulin resistance were significantly reduced. NAC improved cerebral cortex redox status by increasing GSH level and catalase activity while reducing MDA, peroxynitrite, and myeloperoxidase activity. NAC also attenuated neuroinflammation and necroptosis by lowering levels of receptor-interacting protein kinase-3 (RIPK3) and mixed lineage kinase domain-like protein (MLKL), necroptotic signaling molecules that activate the inflammasome, as well as NLRP3 and ASC, an inflammasome component protein. NAC also downregulated pyroptosis-related cytokine IL-18. Histologically, NAC preserved cerebral cortex architecture and reduced astrogliosis, as evidenced by decreased glial fibrillary acidic protein (GFAP), iNOS, and matrix metalloproteinase 9 (MMP-9) immunoreactivity [103].

NAC or taurine administered to female mice fed HFD prevented HFD-induced memory impairment in both novel object and novel location recognition tasks. It also preserved hippocampal levels of N-acetylaspartate, lactate, and the phosphocreatine-to-creatine ratio, which are markers of neuronal health and energy metabolism. Unlike taurine, NAC increased hippocampal concentrations of glutamate, glutamine, GABA, and glutathione [137]. NAC can also modulate addiction-like behaviors toward high-fat, high-sugar (HFHS) food. Both systemic and intracerebroventricular administration of NAC prior to limited access to a highly palatable Western diet (WD) significantly reduced WD consumption without affecting intake of standard chow, indicating a selective suppression of hedonic feeding rather than general appetite. Importantly, NAC did not induce conditioned taste aversion, suggesting its effects were not due to malaise [138]. Similarly, NAC significantly reduced compulsive-like food-seeking behavior in DIO (diet-induced obesity) rats, particularly persistent lever pressing during reward-unavailable periods, and prevented escalation of HFHS intake. The treatment did not affect general locomotion, anxiety-like behavior, or re-acquisition of operant responding, suggesting specificity for compulsive eating [139].

### 5.8. NAC and Its Role in Cardiovascular Protection

**Figure 4:**
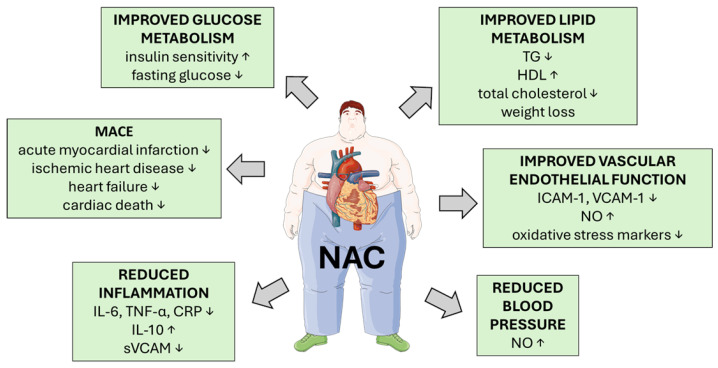
NAC and MetS/obesity related cardiovascular complications.

NAC’s cardioprotective effects are evident in multiple models. It is believed that the mechanism of NAC action in myocardial infarction and HF relies on ROS inhibition, antiplatelet, anti-inflammatory, and anti-apoptotic activity, reduction in obesity-related cardiac insulin resistance, as well as inhibition of angiotensin-converting enzyme (ACE) [140]. Also, NAC may have a beneficial effect on EC dysfunction, which arises from oxidative stress, eNOS uncoupling, leukocyte adhesion, and apoptosis. These processes are exacerbated by MetS components such as hyperglycemia, dyslipidemia, and insulin resistance [141,142]. There are several human studies and clinical trials involving NAC and its use in the mitigation of CVD symptoms, although most of the trials do not involve MetS patients. Nevertheless, it is worth describing these studies since most of the enrolled individuals suffer from one or more MetS symptoms, such as obesity, hypertension, and atherosclerosis (Summarized in Table 3). NAC enhances intracellular GSH levels, reducing oxidative stress and inflammation. In the comprehensive review by Xu et al. [142], NAC is highlighted as a potent antioxidant with therapeutic potential in mitigating endothelial dysfunction caused by oxidative stress. Oxidative stress is a key driver of endothelial dysfunction in MetS, contributing to impaired nitric oxide (NO) bioavailability, inflammation, and vascular permeability. NAC’s ability to scavenge ROS and restore redox balance directly supports endothelial integrity and function. Moreover, NAC is noted to inhibit leukocyte adhesion, reduce NADPH oxidase (NOX) expression, suppress inflammatory cytokine secretion and improve endothelial barrier function [142] (Figure 4).

NAC supplementation to Wistar rats fed either a standard or HFD significantly reduced body mass, plasma glucose and insulin levels, improving insulin sensitivity, but also lowered ceramide accumulation in cardiac tissue by downregulating key enzymes involved in ceramide synthesis, including serine palmitoyltransferase long chain base subunit 2 (SPTLC2), ceramide synthase 5 (LASS5), neutral ceramidase (ASAH2), alkaline sphingomyelinase (Alk-SMase), and neutral sphingomyelinase (N-SMase). This reduction in ceramide was accompanied by an increase in S1P levels and an elevated S1P/ceramide ratio, indicating a shift toward a more protective, anti-apoptotic sphingolipid profile. NAC also improved insulin signaling by enhancing phosphorylation of AKT and upregulation of GLUT4 expression in the left ventricle, thereby promoting glucose uptake. These effects were observed both in rats on a standard diet and those subjected to lipid overload via an HFD [99].

In a rat model of diabetic cardiac hypertrophy induced by alloxan, treatment with NAC markedly reduced blood glucose levels, restored body weight, and improved both heart weight-to-body weight and heart weight-to-tibia length ratios. Histological analysis revealed that NAC preserved myocardial architecture, reduced cardiomyocyte hypertrophy, and prevented fibrosis. Moreover, NAC effectively lowered ROS levels and lipid peroxidation, while enhancing antioxidant enzymes activation, including SOD and catalase. At the molecular level, NAC downregulated pro-apoptotic markers such as dynamin-related protein 1 (Drp1), p53-upregulated modulator of apoptosis (PUMA), and cytochrome c. Concurrently, it upregulated anti-apoptotic and mitochondrial maintenance genes, including Bcl-2 and mitofusin-2 (MFN-2) mitochondrial fusion marker. Additionally, NAC improved lipid profiles by reducing serum TG and cholesterol [143]. Unfortunately, human studies do not show the beneficial effects of NAC on cardiac architecture. In HALT-HCM (Hypertrophy Regression With N-Acetylcysteine in Hypertrophic Cardiomyopathy), a randomized, double-blind, placebo-controlled pilot study, 42 patients with MetS comorbidities such as hypertension, dyslipidemia, and diabetes, with established hypertrophic cardiomyopathy (HCM), received high-dose NAC or placebo for 12 months, and the study found only negligible to modest effects on echocardiographic and cardiac MRI indices of hypertrophy and fibrosis. Moreover, no significant improvements in clinical symptoms or exercise tolerance were observed to evaluate the effects of NAC. Since this was a pilot study with the risk of bias calculated as moderate to high due to the small sample size and exploratory design, more and better conducted clinical trials are necessary to evaluate NAC’s impact on cardiac architecture [144].

Some data show that NAC may prevent diastolic dysfunction in. The treatment of HFHS-fed rats with NAC inhibited the rise in E/DT ratio, an echocardiographic marker of diastolic dysfunction and elevated end-diastolic pressure, without reversing right ventricular hypertrophy [145]. In the estrogen-deprived obese insulin-resistant female rat model, NAC monotherapy partially improved metabolic parameters, including reductions in plasma glucose TG, but did not significantly improve insulin resistance or cardiac function. However, when combined with low-dose estrogen, NAC showed efficacy comparable to regular-dose estrogen in improving left ventricular function, heart rate variability, and reducing cardiac ischemia-reperfusion (I/R) injury. The low-dose estrogen with NAC reduced oxidative stress markers like MDA and increased anti-apoptotic Bcl-2 expression, though it did not suppress pro-apoptotic Bax as effectively as regular-dose estrogen [146]. Treatment with NAC and allopurinol (ALP) in the diabetic rat model synergistically mitigated cardiac damage. NAC and ALP increased cardiac adiponectin and its receptor AdipoR2 expression, restored phosphorylation of key survival pathways—PI3K/AKT and Jak2/STAT3—and upregulated eNOS activation. These molecular changes correlated with reduced infarct size and lower creatine kinase-MB (CK-MB) release [114].

NAC also showed anti-atherosclerotic effects in diabetic ApoE^−^/^−^ mice, which exhibit elevated levels of methylglyoxal (MG), a key precursor in the formation of AGEs and a marker of carbonyl stress. Following 12 weeks of NAC treatment, MG levels were significantly reduced. NAC also restored GSH levels in the aorta, thereby enhancing MG elimination and reducing oxidative stress markers such as serum MDA, and upregulated antioxidant enzyme expression, including SOD-1 and GPX-1. Endothelial function was improved, as evidenced by increased phosphorylation of AKT and eNOS, along with elevated serum NO levels [102]. NAC seems to delay cellular senescence in ECs isolated from patients with severe CAD by reducing oxidative stress and activating telomerase (hTERT), leading to telomere stabilization. NAC also decreased lipid peroxidation, lowered DNA damage markers (ATM, H2AX, p53, promyelocytic leukemia protein—PML). These molecular changes were accompanied by extended cell replication and delayed onset of senescence [147].

NAC lowers plasma homocysteine (Hcy) levels, a known risk factor for MetS. Increased levels of Hcy are associated with CVD, stroke, and obesity, although some authors underline iatrogenic elevation of Hcy in MetS, since most hypolipidemic, antidiabetic, and antihypertensive drugs raise circulating Hcy levels [148]. NAC may reduce Hcy blood levels by increasing urinary excretion, as was shown in a single-arm interventional study. Intravenous NAC administration to healthy individuals led to a rapid and significant reduction in plasma Hcy levels, which was accompanied by a marked increase in urinary excretion, and the mechanism was thought to involve NAC displacing thiols from protein-binding sites, forming mixed disulphides that are more readily cleared renally. Unfortunately, the risk of bias for this study was estimated as moderate due to the small sample size, lack of control group, and short duration [149]. A double-blind, randomized, placebo-controlled crossover trial investigated the effects of oral NAC on plasma Hcy and lipoprotein levels in patients with elevated cardiovascular risk markers. Eleven subjects with high plasma lipoprotein levels were recruited, and NAC was administered at a high dose over a two-week period. While NAC had no significant effect on lipoprotein levels, it caused a substantial reduction in plasma Hcy. The treatment was well tolerated, with only mild side effects like flatulence and bad taste, and the risk of bias for this study was estimated as low due to rigorous design, validated assays, and appropriate statistical analysis [150]. Hildebrandt et al. [151] reanalyzed two randomized, double-blind, placebo-controlled trials to assess the long-term effects of oral NAC on plasma Hcy and blood pressure in normolipidemic and hyperlipidemic men, stratified by smoking status. NAC supplementation significantly reduced plasma Hcy concentrations, independent of lipid profile or smoking status. This reduction was accompanied by increased plasma cysteine levels and improved intracellular redox status, particularly in smokers. NAC also significantly lowered systolic blood pressure in both groups, with a notable reduction in diastolic pressure observed only in hyperlipidemic subjects [151]. Supporting these findings, a comprehensive meta-analysis of 28 studies confirmed that NAC significantly reduces Hcy levels, as well as oxidative stress markers such as MDA and proinflammatory cytokines, including IL-8, IL-6, and TNF-α [152].

NAC may also stabilize levels of NO, since it supplies free sulfhydryl groups. This prevents NO degradation and enhances vasodilation, and finally improves vascular endothelial function and reduces nitrate tolerance [140]. In a randomized, double-blind, placebo-controlled, four-arm trial, NAC was evaluated for its potential to enhance the long-term treatment of unstable angina pectoris, since it is known to potentiate the vasodilatory effects of nitroglycerin and prevent nitrate tolerance. Although the inclusion criteria did not include MetS, but unstable angina, some of the individuals exhibited MetS symptoms, such as diabetes and hypertension. The combination of nitroglycerin and NAC significantly reduced the incidence of adverse cardiac events (death and MI), presumably due to enhanced NO bioavailability and antiplatelet effects. However, the combination therapy was associated with a higher incidence of severe headaches, leading to more frequent drug discontinuation, although the risk of bias for this study was estimated as low, due to robust design, standardized protocols, and good compliance [153]. NAC improves blood pressure control not only by enhancing NO bioavailability, but also by inhibiting ACE. Clinical studies also showed reductions in systolic and diastolic pressure in patients with T2DM and hypertension. There was a randomized, double-blind, placebo-controlled trial to evaluate the effects of NAC and L-arginine (ARG) on endothelial function in hypertensive male patients with T2DM. The study focused on NO bioavailability, oxidative stress, and vascular health. The NAC+ARG group showed significant reductions in systolic and diastolic blood pressure, total and LDL cholesterol, oxidized LDL, CRP, nitrotyrosine, biomarker of nitrosative stress, fibrinogen, and adhesion molecules (ICAM-1, VCAM-1, and PAI-1). These changes were accompanied by improved intima-media thickness and increased plasma nitrites/nitrates levels. Unfortunately, the risk of bias for this study was estimated to be moderate due to the small sample size and short duration, despite rigorous design and comprehensive biomarker analysis [154]. In an open-label, within-patient crossover study, NAC significantly reduced both 24-hour and daytime systolic and diastolic blood pressure compared to the ACE inhibitor (captopril or enalapril) alone in hypertensive smokers. In this study, the patients were not suffering from MetS, although hypertension is one of its symptoms, and therefore results may translate into such populations. The effect was attributed to NAC’s function as a sulfhydryl donor, enhancing NO bioavailability and protecting it from oxidative degradation. The risk of bias for this study was estimated as moderate due to the small sample size, short duration, and open-label design, but the crossover structure strengthened internal validity [155]. As was stated above, NAC also enhances the antiplatelet and hemodynamic effects of nitrates and reduces nitrate tolerance. Therefore, it may improve outcomes in patients with coronary artery disease, having overlapping risk factors such as hypertension, diabetes, and hyperlipidemia [156].

Several studies support NAC’s role in reducing oxidative stress and improving cardiac function in major adverse cardiac events (MACEs). Although MetS was not explicitly studied in most of these trials, many participants had overlapping risk factors such as hypertension, diabetes, and hyperlipidemia. Population-based cohort study with propensity score matching used Taiwan’s National Health Insurance Research Database to evaluate the long-term effects of NAC on MACE in patients with T2DM. The study included 46,718 T2DM patients, with 23,359 receiving long-term NAC therapy and 23,359 matched controls. NAC users exhibited a significantly lower incidence of MACE compared to non-users. NAC was associated with reduced risks of acute myocardial infarction, ischemic heart disease, cerebrovascular accidents, HF, and cardiac death, although the risk of bias for this study was estimated to be moderate due to the observational design that limits causal inference [157]. A double-blind, randomized, placebo-controlled clinical trial involved ninety-eight patients with ST-elevation myocardial infarction (STEMI) who received either NAC or placebo for three days. NAC significantly reduced serum levels of MMP-2 and MMP-9 after 72 hours. Patients treated with NAC also had a lower incidence of MACE at one-year follow-up, including fewer reinfarctions, but the small sample size and short NAC exposure reduce the robustness of the results [158]. A controlled, non-randomized clinical trial involving patients with acute myocardial infarction (AMI) treated with streptokinase demonstrated that intravenous NAC significantly reduced plasma MDA levels and improved left ventricular (LV) function, including ejection fraction and wall motion, despite no change in infarct size, but due to the small sample size and short NAC exposure, outcomes are subject to bias [159]. Similar results were obtained in a randomized, open-label, safety-focused pilot trial with AMI patients treated with streptokinase and nitroglycerin combined with NAC, carrying a comparable risk of bias [160]. A randomized, controlled, open-label pilot study reported reduced lipid hydroperoxides and transient neutrophil suppression with NAC while investigating the effects of NAC as adjunct therapy in patients with AMI undergoing fibrinolytic treatment with streptokinase. NAC administration significantly reduced plasma lipid hydroperoxide (ROOH) levels after 24 hours and transiently reduced circulating PMN counts. The risk of bias for this study was estimated as moderate due to the small sample and limited endpoints [161]. In LIPSIA-N-ACC (Leipzig Immediate Percutaneous Coronary Intervention Acute Myocardial Infarction N-Acetylcysteine), a randomized, single-blind, placebo-controlled trial, NAC reduced oxidative biomarkers (advanced oxidation protein products and oxidized LDL), but it did not improve clinical outcomes such as infarct size, the incidence of CIN, myocardial salvage index (MSI), or microvascular obstruction in unselected patients with STEMI undergoing primary percutaneous coronary intervention (PCI). The low risk of bias strengthens the validity of the findings [162]. In contrast, the NACIAM (N-Acetylcysteine in Acute Myocardial Infarction), a randomized, double-blind, placebo-controlled, multicenter trial, showed that high-dose NAC combined with low-dose nitroglycerin significantly reduced infarct size and enhanced myocardial salvage in patients with STEMI undergoing primary PCI, with sustained benefits at 3 months. NAC treatment resulted in a significant absolute reduction in infarct size by 5. 5% compared to placebo, and late cardiac magnetic resonance (CMR) imaging at 3 months confirmed sustained infarct size reduction. This study also demonstrates a low risk of bias, making its findings more reliable [163].

The results from human studies are summarized in Table 3.

**Table 3:** Human studies involving NAC use in management of MetS and its comorbid conditions.

Design	Population	Dose/Route	Duration	Comparators	Effect Sizes	Safety	Risk of Bias
Randomized, double-blind, placebo-controlled clinical trial [127]	40 obese adults (BMI ≥ 35 kg/m^2^), aged 25–50, candidates for bariatric surgery	600 mg/day oral NAC	4 weeks before bariatric surgery	Placebo	**Adipose tissue sample**: SA-β-gal ↓ staining (*p* = 0.001), IL-6 ↓ (*p* = 0.014), P16 ↓ (*p* = 0.047) gene expression **Blood:** hsCRP, IL-6 FSB, insulin ↓ (*p* < 0.001) **HOMA-IR** ↓ (*p* < 0.001)	No adverse events reported	Low
Randomized, double-blind, placebo-controlled clinical trial [128]	24 older adults (61–80 years, BMI > 27), 12 young adults (21–40 years)	GlyNAC: glycine (100 mg/kg/day) + NAC (100 mg/kg/day), oral	16 weeks (YA received GlyNAC for 2 weeks)	Placebo	**Muscle sample:** GSH ↑ (*p* < 0.001) **Blood:** RBC total GSH ↑ (*p* < 0.001), TBARS ↓ (*p* < 0.001), IL-6 ↓ (*p* < 0.001), TNFα ↓ (*p* < 0.001), hsCRP ↓ (*p* < 0.001), IL-10 ↑ (*p* < 0.001), sICAM1 ↓ (*p* < 0.001), sVCAM1 ↓ (*p* < 0.001), **HOMA-IR** ↓ (*p* < 0.001), improved cognition, gait speed	No adverse events reported	Low
Double-blind, randomized, placebo-controlled crossover trial [150]	11 adults (mean age 58), with elevated plasma lipoprotein(a)	2 g NAC twice daily, oral	2 weeks NAC, 2 weeks placebo, with 2-week washout between	Placebo	Homocysteine ↓ (*p* < 0.0001); Cysteinyl glycine ↓ (*p* = 0.0001); Cysteine ↓ (*p* = 0.013)	Well tolerated; minor side effects (flatulence, bad taste)	Low
Randomized, double-blind, placebo-controlled, multicenter trial (NACIAM) [163]	112 STEMI patients undergoing PCI	NAC 29 g IV over 48 h + nitroglycerin 7.2 mg IV over 48 h	48 h treatment; 3-month follow-up	Placebo + nitroglycerin	Infarct size ↓ (*p* = 0.02); Myocardial salvage ↑ (*p* < 0.01); Late infarct size ↓ (*p* = 0.02)	No increase in hypotension, bleeding, or nephropathy; 2 deaths in placebo group, none in NAC group	Low
Randomized, double-blind, placebo-controlled, 4-arm trial [153]	200 patients with unstable angina not requiring emergency revascularization	NAC 600 mg orally, 3× daily; Nitroglycerin 10 mg transdermal patch daily	4 months	Placebo, Nitroglycerin alone, NAC alone, Nitroglycerin + NAC	Combined therapy reduced outcome events (death, MI, refractory angina): vs. placebo (OR 0.25; 95% CI 0.07–0.7; *p* = 0.0022); vs. Nitroglycerin (OR 0.31; 95% CI 0.098–0.90; *p* = 0.018); vs. NAC (OR 0.19; 95% CI 0.06–0.57; *p* = 0.0008)	High incidence of severe headache in combined group (31% vs. 19% in Nitroglycerin alone); 33% discontinued due to side effects	Low
Randomized, single-blind, placebo-controlled trial (LIPSIA-N-ACC) [162]	251 STEMI patients undergoing PCI	NAC 6 g IV over 48 h (1.2 g bolus + 2 × 1.2 g/day)	48 h treatment; 6-month follow-up	Placebo + hydration	No significant difference in CIN, oxidative stress markers ↓ (*p* < 0.05)	No adverse events	Low
Open-label clinical trial [88]	8 OA (71–80 years), compared to 8 YA (21–30 years)	GlyNAC: glycine (1.33 mmol/kg/day) + NAC (0.81 mmol/kg/day), oral	24 weeks supplementation + 12 weeks withdrawal	Young adults (baseline comparison)	**Blood:** RBC-reduced GSH ↑ (*p* = 0.0000) TBARS ↓ (*p* = 0.0000), IL-6 ↓ (*p* = 0.0000), TNF-α ↓ (*p* = 0.0000), hsCRP ↓ (*p* = 0.0000), IL-10 ↑ (*p* = 0.0000), sICAM1 ↓ (*p* = 0.0000), sVCAM1 ↓ (*p* = 0.0002), glucose ↓ (*p* = 0.04), **HOMA-IR** ↓ (*p* = 0.0000), improved cognition, gait speed	No adverse events reported	Moderate
Randomized controlled pilot trial [90]	27 adults (45–65 years) with MetS and at risk of MASLD	MetioNac^®^: 3 capsules/day (each capsule: SAMe 200 mg, NAC 100 mg, thioctic acid 75 mg, vitamin B6 0.65 mg), oral	3 months	Control: semipersonalized MD	**Blood:** TG ↓ (*p* = 0.043), VLDL ↓ (*p* = 0.048)	No adverse events reported	Moderate
Randomized, double-blind, placebo-controlled trial [154]	24 male patients with T2DM and hypertension	NAC 600 mg orally twice daily + L-arginine 1200 mg orally once daily	6 months	Placebo	Significant reductions in SBP and DBP (*p* = 0.05), total cholesterol (*p* = 0.01), LDL (*p* = 0.005), ox-LDL (*p* = 0.05), hsCRP (*p* = 0.05), sICAM (*p* = 0.05), sVCAM (*p* = 0.01)	No adverse effects	Moderate
Population-based cohort study with propensity score matching [157]	46,718 patients with T2DM	Oral NAC; average daily dose ≥ 600 mg	Up to 13 years (2008–2021)	Non-NAC users	Overall MACE: average dose aHR 0.84 (95% CI: 0.81–0.86, *p* < 0.0001); highest dose aHR 0.61 (95% CI: 0.58–0.64, *p* < 0.0001)	No adverse effects reported	Moderate
Open-label, within-patient crossover study [155]	18 hypertensive smokers (15 males, 3 females; mean age 69 ± 5 years) on ACEi (captopril or enalapril)	NAC 600 mg orally three times daily (1800 mg/day) added to ACEi	21 days per treatment arm (ACEi alone vs. ACEi + NAC), with 5-day washout	ACEi alone	24 h SBP ↓ (*p* < 0.05); 24 h DBP ↓ (*p* = 0.01); Daytime SBP and DBP ↓ (both *p* < 0.05)	No adverse effects reported	Moderate
Double-blind, randomized, placebo-controlled clinical trial [158]	98 patients with STEMI	NAC 600 mg twice daily, oral	3 days (acute phase), with 1-year follow-up for MACE	Placebo	**72 h**: MMP-9 ↓ (*p* = 0.014); MMP-2 ↓ (*p* = 0.045); **MACE**: 14% (NAC) vs. 25% (placebo), *p* = 0.024; **Reinfarction**: 4% vs. 16.7%, *p* = 0.007	No adverse effects reported	Moderate
Controlled, non-randomized clinical trial [159]	30 patients with acute MI, admitted within 6 h of symptom onset	NAC 15 g IV over 24 h, combined with streptokinase and nitroglycerin	24 h for oxidative stress markers; 3 months for echocardiographic follow-up	Streptokinase + nitroglycerin	MDA ↓ at 4 h and 24 h in NAC group (*p* < 0.01); LVEF ↑ at day 3 and month 3 (*p* < 0.05); LVESD ↓ at day 3 and month 3 (*p* < 0.001); WMSI ↓ at day 3 and month 3 (*p* < 0.05)	No adverse effects reported	Moderate
Randomized, open-label, safety-focused pilot trial [160]	27 AMI patients treated with streptokinase and nitroglycerin	NAC 15 g IV over 24 h	24 h treatment; 7-day follow-up	Streptokinase + nitroglycerin	GSH:GSSG ratio ↑ (*p* < 0.05); MDA ↓ over first 8h (*p* < 0.001); Cardiac index ↑ (*p* = 0.009)	No deaths; minor bleeding in 3 NAC patients; headache in 4; no hypotension	Moderate
Randomized, controlled, open-label pilot study [161]	16 AMI patients (mean age 52)	NAC 15 g IV over 24 h + nitroglycerin + streptokinase	48 h treatment; 6-month follow-up	Nitroglycerin + streptokinase	Plasma hydroperoxides ↓ at 24 h (*p* = 0.039); PMN count ↓ at 6–24 h (*p* < 0.01)	No adverse events	Moderate
Randomized, double-blind, placebo-controlled pilot study [144]	42 adults with HCM, LV wall thickness ≥ 15 mm	NAC: 600 mg twice daily for 3 months, then 1200 mg twice daily for 9 months, oral capsules	12 months	Placebo	LV mass index: Δ = 5.99 g/m^2^ (95% CI: −19.10, 31.10), wall thickness: Δ = 0.79 mm (95% CI: −2.97, 1.39)	6 serious adverse events in NAC group (e.g., pneumonia, CVA, seizure), none attributed to NAC	Moderate to high

Abbreviations: NAC: N-acetylcysteine; BMI: body mass index; SA-β-gal: senescence associated β-galactosidase; IL-6: interleukin 6; hsCRP: high sensitivity C-reactive protein; FSB: fasting blood sugar; HOMA-IR: Homeostatic Model Assessment of Insulin Resistance; OA: older adult; YA: young adult; RBC: red blood cell; GSH: glutathione; TNF-α: tumor necrosis factor alpha; sICAM: soluble intercellular adhesion molecule-1; sVCAM: soluble vascular cell adhesion molecule; TBARS: thiobarbituric acid-reactive substances; MetS: metabolic syndrome; MASLD: metabolic dysfunction-associated steatotic liver disease; SAMe: S-adenosyl-L-methionine; TG: triglycerides; VLDL: very low-density lipoprotein; HCM: hypertrophic cardiomyopathy; LV: left ventricle; CVA: cerebrovascular accident; MI: myocardial infarction; MD: mediterranean diet; T2DM: Type 2 Diabetes Mellitus; SBP: systolic blood pressure; DBP: diastolic blood pressure; LDL: low-density lipoprotein; ox-LDL: oxidized low-density lipoprotein; ACEi: angiotensin-converting enzyme inhibitor; MACE: major adverse cardiac events; OR: odds ratio; aHR: adjusted hazard ratio; STEMI: St-elevation myocardial infarction; MDA: malondialdehyde; LVESD: Left Ventricular End-Systolic Diameter; WMSI: Wall Motion Scoring Index; AMI: acute myocardial infarction; GSH:GSSG: glutathione/oxidised glutathione; PMN: polymorphonuclear neutrophil; CIN: contrast-induced nephropathy; PCI: percutaneous coronary intervention.

## 6. Concluding Remarks, Clinical Implications, and Limitations

NAC has emerged as a promising adjunctive agent in the therapeutic management of MetS and its associated comorbidities, including TM2D and CVD. Through replenishment of intracellular GSH and modulation of redox-sensitive signaling pathways, NAC exerts beneficial effects on insulin sensitivity, lipid metabolism, and chronic inflammation. Additionally, its capacity to enhance endothelial function, reduce oxidative stress, and improve lipid profiles underscores its potential in mitigating cardiovascular complications.

From a clinical perspective, NAC’s favorable safety profile, accessibility, and broad spectrum of biological activity support its integration into existing treatment strategies, particularly in patients with early-stage metabolic dysfunction or multiple risk factors. The context-dependent nature of its effects—such as stress reduction under specific metabolic conditions—highlights the importance of individualized dosing, timing, and patient selection. Furthermore, combination therapies involving NAC, such as GlyNAC or MetioNac^®^, may offer enhanced efficacy, especially in aging populations or those with complex metabolic profiles.

Despite these encouraging findings, several limitations must be acknowledged. The majority of available data derive from preclinical studies or small-scale clinical trials, which restrict the generalizability of results. Heterogeneity in study design, dosage, and treatment duration complicates direct comparisons and consensus on optimal therapeutic protocols. Moreover, long-term safety data and potential interactions with standard pharmacotherapies remain insufficiently explored.

To establish NAC’s definitive role in clinical practice, future research should prioritize large, well-controlled randomized trials. These studies should aim to standardize dosing regimens, evaluate long-term outcomes, and define NAC’s place within personalized treatment frameworks. Until such evidence is available, NAC should be considered a valuable adjunct rather than a standalone therapy in the management of MetS and its cardiovascular complications.

## Figures and Tables

**Table 1:** NAC and the modulation of metabolic pathways in the management of MetS and its comorbid conditions.

Metabolic Pathways	Physiological Function	Effect of Dysregulation	Impact of NAC	Model/Dosage
**Lipid** **metabolism**	Fatty acid oxidation, energy balance	Dyslipidemia, ectopic fat accumulation	Increases fatty acid oxidation, reduces lipogenesis, normalizes lipid profiles	cultured 3T3-L1 preadipocytes, 10 μM NAC [92] BMAT in vitro, 10 mM NAC [93] Wistar rats injected AGE-albumin, NAC in drinking water (600 mg/L) [94] HFD rat, 500 mg/kg NAC [95]
**Glucose metabolism**	Maintaining energy homeostasis via ATP production, glucose synthesis, redox balance, and storage	Dysregulated glucose flux, lactate accumulation, oxidative stress, insulin resistance	Improves insulin signaling, reduces oxidative stress, enhances glycogen synthesis, modulates glycolysis, improves glucose tolerance and uptake	3T3-L1 murine fibroblasts, 2.5 or 5 mM NAC [96] iFIRKO mice, 15 mmol/L NAC in drinking water [87] HFD mice, 50 mg/kg NAC [97]
**Insulin signaling**	Glucose uptake, lipid metabolism	Insulin resistance, hyperglycemia	Improves insulin sensitivity, reduces inflammation in adipose tissue	3T3-L1 fibroblasts, 50 μM NAC [98] HFD rat, 500 mg/kg NAC [99] HFD mice, 50 mg/kg NAC [100]
**Oxidative stress response**	ROS detoxification, redox balance	Increased accumulation of ROS—endothelial dysfunction, mitochondrial damage	Increases GSH, scavenges ROS, protects mitochondria and vascular endothelium	3T3-L1 adipocytes, 1.0 mmol/L NAC [101] ApoE-/- mice, 2 mmol/L NAC in drinking water [102] HFD rat, 150 mg/kg NAC [103]
**Metaflammation**	Immune regulation, tissue repair	Chronic low-grade Inflammation, elevated proinflammatory cytokines such tnf-α, il-6	Reduces pro-inflammatory cytokines, attenuates chronic low-grade inflammation, improves adiponectin levels	human ATM, 5 mM NAC [104] 3T3-L1 adipocytes, 1 mM NAC [105] HFD rat, 500 mg/kg NAC [106]
**Mitochondrial metabolism**	Energy production, apoptosis regulation	Mitochondrial dysfunction, reduced membrane potential	Improves energy metabolism and prevents mitochondrial dysfunction in adipose tissue and cardiomyocytes, restores mitochondrial membrane potential	pancreatic-βcells, 10 mM NAC [107] HFD mice, 60 mg/kg NAC [108]
**Molecular hallmarks of aging**	Increase in activity with age	Increase in sa–β-gal and p16, p21 expression	Reduces SA–β-gal activity and expression of p16, p21	pol η−/− mice, 1 mg/mL NAC in drinking water [109]
**Apoptosis**	Cell growth, metabolism, and survival	Hypertrophy and apoptosis of cardiomyocytes	Reduces oxidative stress-induced hypertrophy and cardiomyocyte apoptosis	pancreatic-βcells, 10 mM NAC [107] NRCM cardiomyocytes, 100 µM NAC [75]

Abbreviations: NAC: N-acetylcysteine; MetS: metabolic syndrome; PI3K: phosphoinositide 3-kinase; ROS: reactive oxygen species; GSH: glutathione; TNF-α: tumor necrosis factor alpha; IL-6: interleukin 6; SA–β-gal: senescence-associated beta-galactosidase; ER: endoplasmic reticulum; NRCM: primary cultured neo-natal rat cardiomyocytes; HFD: high-fat diet.

**Table 2:** NAC-mediated modulation of cellular signaling in obesity/MetS-related cardiometabolic disorders.

Cellular Signaling	Physiological Role	Effect of Dysregulation	Impact of NAC	Model/Dosage
**AMPK**	Energy sensor, catabolic pathways activation at low ATP	Impaired energy balance, lipid accumulation	Indirectly supports AMPK activation, restores redox balance,	pancreatic-βcells, 10 mM NAC [107] HFD mice, 60 mg/kg NAC [108]
**m-TOR**	Cell growth, protein synthesis regulation, and nutrient sensing	Lipogenesis promotion, insulin resistance	Reduces oxidative stress, may normalize mTOR signaling	pancreatic-βcells, 10 mM NAC [107] HFD rat, 500 mg/kg NAC [110]
**ROS/NRF2**	cellular resistance to oxidation regulation	Inflammation, cellular damage	Boosts glutathione, activates NRF2, reduces ROS	3T3-L1 murine fibro-blasts, 2.5 or 5 mM NAC [96] HFD mice, 0.5, 2 or 10 g/L NAC in drinking water [111]
**ROS–NLRP3**	Inflammatory response triggered by mitochondrial DAMPs	Chronic inflammation and tissue injury, insulin resistance in skeletal muscle	Inhibits NLRP3 activation by lowering ROS restored insulin-dependent glucose uptake	HFD rat, 150 mg/kg NAC [103] HFD diabetic rat, 20 mg/kg NAC [112]
**PI3K/AKT**	Insulin signaling mediation, glucose uptake regulation	Insulin resistance, hyperglycemia, metabolic imbalance, oxidative stress and inflammation increase, mitochondrial dysfunction and apoptosis of cardiomyocytes, pathological cardiac remodeling and progression to HF	Improves insulin sensitivity by reducing oxidative and ER stress, enhances AKT expression, elevates phosphorylated AKT levels	NRCM cardiomyocytes, 100 µM NAC [75] 3T3-L1 fibroblasts, 50 μM NAC [98] HFD mice, 50 mg/kg NAC [97] HFD mice, 50 mg/kg NAC [100]
**SIRT1/** **SIRT3**	Mitochondrial function oxidative stress, and metabolism regulation	Mitochondrial dysfunction and ROS accumulation	Increases the NAD^+^/NADH ratio, supports SIRT1/3 activity, reduces inflammation	BMAT in vitro, 10 mM NAC [93]
**NFκB/** **AP-1/** **JNK**	Inflammatory response regulation	Chronic inflammation; insulin resistance, endothelial dysfunction	Inhibits NFκB-mediated inflammation	3T3-L1 adipocytes, 1 mM NAC [105] HFD rat, 500 mg/kg NAC [110]
**MAPK (ERK/JNK)**	Cellular proliferation, differentiation, and stress response regulation	Adipogenesis and inflammation activation	Inhibits ERK1/2 and JNK1/2 phosphorylation; suppresses adipocyte differentiation and inflammatory signaling	3T3-L1 preadipocytes, 0.01 or 1 mM NAC [113]
**STAT3**	Cytokine signaling and immune response regulation	Chronic inflammation and metabolic dysfunction activation	Inhibits STAT3 activation via antioxidant and anti-inflammatory mechanisms	HFD diabetic rat, 1.5 g/kgNAC [114]
**GSK3β**	Glycogen synthesis and insulin signaling regulation	Altered glucose metabolism and defective insulin signaling	Restores GSK3β phosphorylation; improves insulin signaling	HFD rat, 500 mg/kg NAC [110] T3-L1 preadipocytes, 100 µM NAC [115]
**IRS1/PI3K**	Insulin signaling cascade initiation	Glucose uptake dysfunction, insulin resistance	Enhances IRS1/PI3K association, improves insulin signalling	HFD mice, 50 mg/kg NAC [97]
**CHOP**	ER stress marker, apoptosis promotion	ER stress, mitochondrial dysfunction, inflammatory activation	Reduces CHOP accumulation; restores anti-inflammatory cytokine secretion	3T3-L1 murine fibroblasts, 2.5 or 5 mM NAC [96]
**UCP1/** **PGC-1α**	Thermogenic response and mitochondrial biogenesis induction	Lowered energy output and adiposity development	Induces browning of WAT via NRF2/HO-1 axis; upregulates UCP1 and PGC-1α	HFD mice, 400 mg/kg NAC (with other cofactors) [89] HFD mice, 0.5, 2, or 10 g/L NAC in drinking water 111 [111]

Abbreviations: NAC: N-acetylcysteine; AMPK: AMP-activated protein kinase; mTOR: mechanistic target of rapamycin; ROS: reactive oxygen species; HF: heart failure; NRF2: nuclear factor erythroid 2–related factor 2; NLRP3: NOD-, LRR- and pyrin domain-containing protein 3; PI3K: phosphoinositide 3-kinase; AKT: protein kinase B; ER: endoplasmic reticulum; SIRT1/SIRT3: sirtuin 1/sirtuin 3; NAD^+^/NADH: nicotinamide adenine dinucleotide (oxidized/reduced forms); NF-κB: nuclear factor kappa-light-chain enhancer of activated B cells; AP-1: activator protein 1: JNK: c-Jun N-terminal kinase; STAT: signal transducer and activator of transcription 3; MAPK: mitogen-activated protein kinase; ERK: extracellular signal-regulated kinase; GSK3β: glycogen synthase kinase 3 beta; IRS1: insulin receptor substrate 1; CHOP: C/EBP homologous protein; UCP1: uncoupling protein 1; HO-1: heme oxygenase 1; PPARγ: peroxisome proliferator-activated receptor gamma; C/EBPβ: CCAAT/enhancer binding protein beta.

## Data Availability

No new data were created or analyzed in this study.

## Abbreviations

The following abbreviations are used in this manuscript:

WHO	World Health Organisation
Mets	Metabolic Syndrome
LAP	Lipid Accumulation Product
CMI	Cardiometabolic Index
BMI	Body Mass Index
Cvd	Cardiovascular Disease
Nac	N-Acetylcysteine
Masld	Metabolic Dysfunction-Associated Steatotic Liver Disease
HF	heart failure
Ppar	Peroxisome Proliferator-Activated Receptor
Fgf21	Fibroblast Growth Factor 21
Glp-1	Glucagon-Like Peptide-1
Sat	Subcutaneous Adipose Tissue
Vat	Visceral Adipose Tissue
Ffa	Free Fatty Acid
Eat	Epicardial Adipose Tissue
Tg	Triglycerides
HDL	High-Density Lipoprotein
LDL	Low-Density Lipoprotein
Ros	Reactive Oxygen Species
Hif-1	Hypoxia Inducible Factor 1
Mcp-1	Monocyte Chemoattractant Protein-1
TNF-A	Tumor Necrosis Factor A
Prdx3	Peroxiredoxin-3
Cyslt	Cysteinyl Leukotriene
Ltb4	Leukotriene B4
NF-Κb	Nuclear Factor Kappa-Light-Chain Enhancer Of Activated B Cells
Mapk	Mitogen-Activated Protein Kinase
Pi3k/Akt	Phosphoinositide 3-Kinase/Protein Kinase B
Ampk	Amp-Activated Protein Kinase
Sirt3	Sirtuin 3
Gsh	Glutathione
Ace	Angiotensin Converting Enzyme
Sod	Superoxide Dismutase
Jnk	C-Jun N-Terminal Kinase
Erk	Extracellular Signal-Regulated Kinase
Ap-1	Activator Protein 1
Sapk/Ink	Stress-Activated Protein Kinases/Cyclin-Dependent Kinase Inhibitor
Stats	Signal Transducers And Activators Of Transcription
8-Oh-G	8-Hydroxyguanine
Hdacs	Histone Deacetylases
Hats	Histone Acetyltransferases
Pparγ	Peroxisome Proliferator-Activated Receptor Gamma
C/Ebpβ	CCAAT/Enhancer Binding Protein Beta
Mtorc2	Mechanistic Target Of Rapamycin Complex 2
Er	Endoplasmic Reticulum
Mtupr	Mitochondrial Unfolded Protein Response
Hfd	Hight Fat Diet
Lc3-Ii	Autophagy-Related Protein Light Chain 3
Gpx	Glutathione Peroxidase
Irs1	Insulin Receptor Substrate 1
Pdx-1	Pancreatic-Duodenal Homeobox-1
Mda	Malondialdehyde
Gsh/Gssg	Glutathione/Oxidised Glutathione
Sasp	Senescence Secretory Phenotype
CMR	Cardiac magnetic resonance
Tbars	Thiobarbituric Acid-Reactive Substances
Ph2ax	Phospho-H2A Histone Family Member X
Fabp4	Fatty Acid Binding Protein 4
Hsp70	Heat Shock Protein 70
Maoa	Monoamine Oxidase A
Acy-1	Aminoacylase-1
Tkt	Transketolase
Mt3	Metallothionein 3
Mc	Metabolic Cofactor
Homa-Ir	Homeostasis Model Assessment Of Insulin Resistance
Atgl	Adipose Triglyceride Lipase
Plin1	Perilipin 1
Acox1	Acyl-Coa Oxidase 1
Hscrp	High-Sensitivity C-Reactive Protein
Bmat	Bone Marrow Adipose Tissue
Ho-1	Heme Oxygenase-1
Dgat1	Diacylglycerol Acyltransferase
S1p	Sphingosine-1-Phosphate
Sa1p	Sphinganine-1-Phosphate
GSK3α/Β	Glycogen Synthase Kinase-3α/Β
S6rp	S6 Ribosomal Protein
Chop	C/Ebp Homologous Protein
Nrf2	Nuclear Factor Erythroid 2-Related Factor 2
Fat/Cd36	Fatty Acid Translocase
Fabppm	Fatty Acid Binding Protein
Fatp1	Long-Chain Fatty Acid Transport Protein 1
Β-HAD	Β-Hydroxyacyl-Coa Dehydrogenase
Ucp1	Uncoupling Protein 1
PGC1α/Β	Peroxisome Proliferator-Activated Receptor Gamma Coactivator 1-Alpha
Bha	Butylated Hydroxyanisole
Bat	Brown Adipose Tissue
Nox4	Nicotinamide Adenine Dinucleotide Phosphate Oxidase 4
Pa	Palmitate
Grp78	Er Stress Markers 78 Kda Glucose-Regulated Protein
Ire1	Inositol-Requiring Protein 1
VLDL	Very Low-Density Lipoprotein
MitoTEMPO	Mitochondria-Targeted Superoxide Dismutase Mimetic
Pai-1	Plasminogen Activator Inhibitor-1
Inos	Inducible Nitric Oxide Synthase
GSK3β	glycogen synthase kinase 3 beta
Γ-H2AX	H2A Histone Family Member X
Atm	Ataxia-Telangiectasia Mutated
AGE-Albumin	Advanced Glycated Albumin
Aa	Arachidonic Acid
Cox-2	Cyclooxygenase-2
5-Lox	5-Lipoxygenase
Pge2	Prostaglandin E2
Ltc4	Leukotriene C4
Lxa4	Lipoxin A4
4-Hne	4-Hydroxynonenal
Ripk3	Receptor-Interacting Protein Kinase-3
Nlrp3	Nod Like Receptor Protein 3
Mlkl	Mixed Lineage Kinase Domain-Like Protein
Asc	Apoptosis-Associated Speck-Like Proteins
Gfap	Glial Fibrillary Acidic Protein
Mmp-9	Matrix Metalloproteinase 9
Wd	Western Diet
Hfhs	High-Fat, High-Sugar
DIO	diet-induced obesity
Ec	Endothelial Cell
Ace	Angiotensin-Converting Enzyme
No	Nitric Oxide
Nox	Nadph Oxidase
Sptlc2	Serine Palmitoyltransferase Long Chain Base Subunit 2
Lass5	Ceramide Synthase 5
Asah2	Neutral Ceramidase
Alk-Smase	Alkaline Sphingomyelinase
N-Smase	Neutral Sphingomyelinase
Drp1	Dynamin-Related Protein 1
Puma	P53-Upregulated Modulator Of Apoptosis
Mfn-2	Mitofusin-2
Alp	Allopurinol
Mg	Methylglyoxal
Htert	Telomerase
Pml	Promyelocytic Leukemia Protein
Hcy	Homocysteine
Svcam	Soluble Vascular Cell Adhesion Molecule
Arg	L-Arginine
Mace	Major Adverse Cardiac Events
AMI	Acute Myocardial Infarction
Stemi	St-Elevation Myocardial Infarction
Rooh	Lipid Hydroperoxide
Pmn	Polymorphonuclear Neutrophil
Cin	Contrast-Induced Nephropathy
Pci	Percutaneous Coronary Intervention
Msi	Myocardial Salvage Index
T2dm	Type 2 Diabetes Mellitus
Pkc	Phosphokinase C
Il	Interleukin
Prdx2	Peroxiredoxin 2
Mtor	Mammalian Target Of Rapamycin
SA-Β-Gal	Senescence Activated Β-Galactosidase

## References

[1] Collaborators G.B.D.A.B. (2025). Global, regional, and national prevalence of adult overweight and obesity, 1990–2021, with forecasts to 2050: A forecasting study for the Global Burden of Disease Study 2021. Lancet.

[2] Pigeot I., Ahrens W. (2025). Epidemiology of metabolic syndrome. Pflug. Arch..

[3] Chen X., Zhao Y., Sun J., Jiang Y., Tang Y. (2024). Identification of metabolic syndrome using lipid accumulation product and cardiometabolic index based on NHANES data from 2005 to 2018. Nutr. Metab..

[4] Kwon J.H., Jang H.Y., Oh M.J., Rho J.S., Jung J.H., Yum K.S., Han J.W. (2011). Association of visceral fat and risk factors for metabolic syndrome in children and adolescents. Yonsei Med. J..

[5] Shuster A., Patlas M., Pinthus J.H., Mourtzakis M. (2012). The clinical importance of visceral adiposity: A critical review of methods for visceral adipose tissue analysis. Br. J. Radiol..

[6] Lee M.J., Kim J. (2024). The pathophysiology of visceral adipose tissues in cardiometabolic diseases. Biochem. Pharmacol..

[7] Silveira Rossi J.L., Barbalho S.M., Reverete de Araujo R., Bechara M.D., Sloan K.P., Sloan L.A. (2022). Metabolic syndrome and cardiovascular diseases: Going beyond traditional risk factors. Diabetes Metab. Res. Rev..

[8] Cesaro A., De Michele G., Fimiani F., Acerbo V., Scherillo G., Signore G., Rotolo F.P., Scialla F., Raucci G., Panico D. (2023). Visceral adipose tissue and residual cardiovascular risk: A pathological link and new therapeutic options. Front. Cardiovasc. Med..

[9] Lopaschuk G.D. (2017). Metabolic Modulators in Heart Disease: Past, Present, and Future. Can. J. Cardiol..

[10] Rehberger-Likozar A., Sebestjen M. (2015). Influence of trimetazidine and ranolazine on endothelial function in patients with ischemic heart disease. Coron. Artery Dis..

[11] Minano S., Gonzalez-Correa C., Moleon J., Duarte J. (2023). Metabolic Modulators in Cardiovascular Complications of Systemic Lupus Erythematosus. Biomedicines.

[12] Byrne C.D., Targher G., Tilg H. (2024). Thyroid hormone receptor-beta agonists: New MASLD therapies on the horizon. Gut.

[13] Cheng H.S., Tan W.R., Low Z.S., Marvalim C., Lee J.Y.H., Tan N.S. (2019). Exploration and Development of PPAR Modulators in Health and Disease: An Update of Clinical Evidence. Int. J. Mol. Sci..

[14] Loomba R., Sanyal A.J., Kowdley K.V., Bhatt D.L., Alkhouri N., Frias J.P., Bedossa P., Harrison S.A., Lazas D., Barish R. (2023). Randomized, Controlled Trial of the FGF21 Analogue Pegozafermin in NASH. N. Engl. J. Med..

[15] Zheng Z., Zong Y., Ma Y., Tian Y., Pang Y., Zhang C., Gao J. (2024). Glucagon-like peptide-1 receptor: Mechanisms and advances in therapy. Signal Transduct. Target. Ther..

[16] Tenorio M., Graciliano N.G., Moura F.A., Oliveira A.C.M., Goulart M.O.F. (2021). N-Acetylcysteine (NAC): Impacts on Human Health. Antioxidants.

[17] Ying W., Sharma K., Yanek L.R., Vaidya D., Schar M., Markl M., Subramanya V., Soleimani S., Ouyang P., Michos E.D. (2021). Visceral adiposity, muscle composition, and exercise tolerance in heart failure with preserved ejection fraction. ESC Heart Fail..

[18] Ibrahim M.M. (2010). Subcutaneous and visceral adipose tissue: Structural and functional differences. Obes. Rev..

[19] Khawaja T., Nied M., Wilgor A., Neeland I.J. (2024). Impact of Visceral and Hepatic Fat on Cardiometabolic Health. Curr. Cardiol. Rep..

[20] Ouchi N., Parker J.L., Lugus J.J., Walsh K. (2011). Adipokines in inflammation and metabolic disease. Nat. Rev. Immunol..

[21] Iacobellis G. (2015). Local and systemic effects of the multifaceted epicardial adipose tissue depot. Nat. Rev. Endocrinol..

[22] Bhat N., Mani A. (2023). Dysregulation of Lipid and Glucose Metabolism in Nonalcoholic Fatty Liver Disease. Nutrients.

[23] Ko C.W., Qu J., Black D.D., Tso P. (2020). Regulation of intestinal lipid metabolism: Current concepts and relevance to disease. Nat. Rev. Gastroenterol. Hepatol..

[24] Kersten S. (2023). The impact of fasting on adipose tissue metabolism. Biochim. Biophys. Acta Mol. Cell Biol. Lipids.

[25] Deng L., Vrieling F., Stienstra R., Hooiveld G.J., Feitsma A.L., Kersten S. (2022). Macrophages take up VLDL-sized emulsion particles through caveolae-mediated endocytosis and excrete part of the internalized triglycerides as fatty acids. PLoS Biol..

[26] Schade D.S., Shey L., Eaton R.P. (2020). Cholesterol Review: A Metabolically Important Molecule. Endocr. Pract..

[27] Gutierrez D.A., Puglisi M.J., Hasty A.H. (2009). Impact of increased adipose tissue mass on inflammation, insulin resistance, and dyslipidemia. Curr. Diab Rep..

[28] Bentzon J.F., Otsuka F., Virmani R., Falk E. (2014). Mechanisms of plaque formation and rupture. Circ. Res..

[29] Dronkers J., van Veldhuisen D.J., van der Meer P., Meems L.M.G. (2024). Heart Failure and Obesity: Unraveling Molecular Mechanisms of Excess Adipose Tissue. J. Am. Coll. Cardiol..

[30] Trandafir L.M., Dodi G., Frasinariu O., Luca A.C., Butnariu L.I., Tarca E., Moisa S.M. (2022). Tackling Dyslipidemia in Obesity from a Nanotechnology Perspective. Nutrients.

[31] Bays H.E., Toth P.P., Kris-Etherton P.M., Abate N., Aronne L.J., Brown W.V., Gonzalez-Campoy J.M., Jones S.R., Kumar R., La Forge R. (2013). Obesity, adiposity, and dyslipidemia: A consensus statement from the National Lipid Association. J. Clin. Lipidol..

[32] Han H.S., Kang G., Kim J.S., Choi B.H., Koo S.H. (2016). Regulation of glucose metabolism from a liver-centric perspective. Exp. Mol. Med..

[33] Satoh T. (2014). Molecular mechanisms for the regulation of insulin-stimulated glucose uptake by small guanosine triphosphatases in skeletal muscle and adipocytes. Int. J. Mol. Sci..

[34] Bonora M., Patergnani S., Rimessi A., De Marchi E., Suski J.M., Bononi A., Giorgi C., Marchi S., Missiroli S., Poletti F. (2012). ATP synthesis and storage. Purinergic Signal..

[35] Halse R., Bonavaud S.M., Armstrong J.L., McCormack J.G., Yeaman S.J. (2001). Control of glycogen synthesis by glucose, glycogen, and insulin in cultured human muscle cells. Diabetes.

[36] Thomas D.D., Corkey B.E., Istfan N.W., Apovian C.M. (2019). Hyperinsulinemia: An Early Indicator of Metabolic Dysfunction. J. Endocr. Soc..

[37] Arsenault B.J., Carpentier A.C., Poirier P., Despres J.P. (2024). Adiposity, type 2 diabetes and atherosclerotic cardiovascular disease risk: Use and abuse of the body mass index. Atherosclerosis.

[38] Feijoo-Bandin S., Aragon-Herrera A., Morana-Fernandez S., Anido-Varela L., Tarazon E., Rosello-Lleti E., Portoles M., Moscoso I., Gualillo O., Gonzalez-Juanatey J.R. (2020). Adipokines and Inflammation: Focus on Cardiovascular Diseases. Int. J. Mol. Sci..

[39] Clemente-Suarez V.J., Redondo-Florez L., Beltran-Velasco A.I., Martin-Rodriguez A., Martinez-Guardado I., Navarro-Jimenez E., Laborde-Cardenas C.C., Tornero-Aguilera J.F. (2023). The Role of Adipokines in Health and Disease. Biomedicines.

[40] Hemat Jouy S., Mohan S., Scichilone G., Mostafa A., Mahmoud A.M. (2024). Adipokines in the Crosstalk between Adipose Tissues and Other Organs: Implications in Cardiometabolic Diseases. Biomedicines.

[41] Peng J., Chen Q., Wu C. (2023). The role of adiponectin in cardiovascular disease. Cardiovasc. Pathol..

[42] Mellott E., Faulkner J.L. (2023). Mechanisms of leptin-induced endothelial dysfunction. Curr. Opin. Nephrol. Hypertens..

[43] Bays H.E., Kirkpatrick C., Maki K.C., Toth P.P., Morgan R.T., Tondt J., Christensen S.M., Dixon D., Jacobson T.A. (2024). Obesity, dyslipidemia, and cardiovascular disease: A joint expert review from the Obesity Medicine Association and the National Lipid Association 2024. Obes. Pillars.

[44] Tian X., Chen S., Wang P., Xu Q., Zhang Y., Luo Y., Wu S., Wang A. (2022). Insulin resistance mediates obesity-related risk of cardiovascular disease: A prospective cohort study. Cardiovasc. Diabetol..

[45] Luciani L., Pedrelli M., Parini P. (2024). Modification of lipoprotein metabolism and function driving atherogenesis in diabetes. Atherosclerosis.

[46] Cui Y., Zhu Q., Hao H., Flaker G.C., Liu Z. (2023). N-Acetylcysteine and Atherosclerosis: Promises and Challenges. Antioxidants.

[47] Manna P., Jain S.K. (2015). Obesity, Oxidative Stress, Adipose Tissue Dysfunction, and the Associated Health Risks: Causes and Therapeutic Strategies. Metab. Syndr. Relat. Disord..

[48] Panday S., Talreja R., Kavdia M. (2020). The role of glutathione and glutathione peroxidase in regulating cellular level of reactive oxygen and nitrogen species. Microvasc. Res..

[49] Incalza M.A., D’Oria R., Natalicchio A., Perrini S., Laviola L., Giorgino F. (2018). Oxidative stress and reactive oxygen species in endothelial dysfunction associated with cardiovascular and metabolic diseases. Vasc. Pharmacol..

[50] Dan Dunn J., Alvarez L.A., Zhang X., Soldati T. (2015). Reactive oxygen species and mitochondria: A nexus of cellular homeostasis. Redox Biol..

[51] Inoguchi T., Li P., Umeda F., Yu H.Y., Kakimoto M., Imamura M., Aoki T., Etoh T., Hashimoto T., Naruse M. (2000). High glucose level and free fatty acid stimulate reactive oxygen species production through protein kinase C--dependent activation of NAD(P)H oxidase in cultured vascular cells. Diabetes.

[52] Battineni G., Sagaro G.G., Chintalapudi N., Amenta F., Tomassoni D., Tayebati S.K. (2021). Impact of Obesity-Induced Inflammation on Cardiovascular Diseases (CVD). Int. J. Mol. Sci..

[53] Mirabelli M., Misiti R., Sicilia L., Brunetti F.S., Chiefari E., Brunetti A., Foti D.P. (2024). Hypoxia in Human Obesity: New Insights from Inflammation towards Insulin Resistance-A Narrative Review. Int. J. Mol. Sci..

[54] Longo M., Zatterale F., Naderi J., Parrillo L., Formisano P., Raciti G.A., Beguinot F., Miele C. (2019). Adipose Tissue Dysfunction as Determinant of Obesity-Associated Metabolic Complications. Int. J. Mol. Sci..

[55] Schwartz E.A., Reaven P.D. (2012). Lipolysis of triglyceride-rich lipoproteins, vascular inflammation, and atherosclerosis. Biochim. Biophys. Acta.

[56] Tilg H., Moschen A.R. (2006). Adipocytokines: Mediators linking adipose tissue, inflammation and immunity. Nat. Rev. Immunol..

[57] Mancuso P. (2016). The role of adipokines in chronic inflammation. Immunotargets Ther..

[58] Heath D.F. (1985). Subcellular aspects of the response to trauma. Br. Med. Bull..

[59] Lopaschuk G.D., Karwi Q.G., Tian R., Wende A.R., Abel E.D. (2021). Cardiac Energy Metabolism in Heart Failure. Circ. Res..

[60] Gutierrez-Cuevas J., Sandoval-Rodriguez A., Meza-Rios A., Monroy-Ramirez H.C., Galicia-Moreno M., Garcia-Banuelos J., Santos A., Armendariz-Borunda J. (2021). Molecular Mechanisms of Obesity-Linked Cardiac Dysfunction: An Up-Date on Current Knowledge. Cells.

[61] Zhang W., Elimban V., Nijjar M.S., Gupta S.K., Dhalla N.S. (2003). Role of mitogen-activated protein kinase in cardiac hypertrophy and heart failure. Exp. Clin. Cardiol..

[62] Li J., Sun M., Tang M., Song X., Zheng K., Meng T., Li C., Du L. (2025). Mechanism of PI3K/Akt-mediated mitochondrial pathway in obesity-induced apoptosis (Review). Biomed. Rep..

[63] Wu S., Zou M.H. (2020). AMPK, Mitochondrial Function, and Cardiovascular Disease. Int. J. Mol. Sci..

[64] Sun W., Liu C., Chen Q., Liu N., Yan Y., Liu B. (2018). SIRT3: A New Regulator of Cardiovascular Diseases. Oxid. Med. Cell Longev..

[65] Zhang Q., Siyuan Z., Xing C., Ruxiu L. (2024). SIRT3 regulates mitochondrial function: A promising star target for cardiovascular disease therapy. Biomed. Pharmacother..

[66] Tieu S., Charchoglyan A., Paulsen L., Wagter-Lesperance L.C., Shandilya U.K., Bridle B.W., Mallard B.A., Karrow N.A. (2023). N-Acetylcysteine and Its Immunomodulatory Properties in Humans and Domesticated Animals. Antioxidants.

[67] Hou Y., Wang L., Yi D., Wu G. (2015). N-acetylcysteine and intestinal health: A focus on its mechanism of action. Front. Biosci..

[68] de Andrade K.Q., Moura F.A., dos Santos J.M., de Araujo O.R., de Farias Santos J.C., Goulart M.O. (2015). Oxidative Stress and Inflammation in Hepatic Diseases: Therapeutic Possibilities of N-Acetylcysteine. Int. J. Mol. Sci..

[69] Mlejnek P., Dolezel P., Kriegova E., Pastvova N. (2021). N-acetylcysteine Can Induce Massive Oxidative Stress, Resulting in Cell Death with Apoptotic Features in Human Leukemia Cells. Int. J. Mol. Sci..

[70] Galicia-Moreno M., Monroy-Ramirez H.C., Caloca-Camarena F., Arceo-Orozco S., Muriel P., Sandoval-Rodriguez A., Garcia-Banuelos J., Garcia-Gonzalez A., Navarro-Partida J., Armendariz-Borunda J. (2025). A new opportunity for N-acetylcysteine. An outline of its classic antioxidant effects and its pharmacological potential as an epigenetic modulator in liver diseases treatment. Naunyn Schmiedebergs Arch. Pharmacol..

[71] Ezerina D., Takano Y., Hanaoka K., Urano Y., Dick T.P. (2018). N-Acetyl Cysteine Functions as a Fast-Acting Antioxidant by Triggering Intracellular H(2)S and Sulfane Sulfur Production. Cell Chem. Biol..

[72] Lee S.J., Noh H.J., Sung E.G., Song I.H., Kim J.Y., Kwon T.K., Lee T.J. (2011). Berberine sensitizes TRAIL-induced apoptosis through proteasome-mediated downregulation of c-FLIP and Mcl-1 proteins. Int. J. Oncol..

[73] Gutteridge J.M., Mumby S., Quinlan G.J., Chung K.F., Evans T.W. (1996). Pro-oxidant iron is present in human pulmonary epithelial lining fluid: Implications for oxidative stress in the lung. Biochem. Biophys. Res. Commun..

[74] Yu N., Wu X., Zhang C., Qin Q., Gu Y., Ke W., Liu X., Zhang Q., Liu Z., Chen M. (2024). NADPH and NAC synergistically inhibits chronic ocular hypertension-induced neurodegeneration and neuroinflammation through regulating p38/MAPK pathway and peroxidation. Biomed. Pharmacother..

[75] Chao S.P., Cheng W.L., Yi W., Cai H.H., Deng K., Cao J.L., Zeng Z., Wang H., Wu X. (2024). N-Acetylcysteine Alleviates Phenylephrine-Induced Cardiomyocyte Dysfunction via Engaging PI3K/AKT Signaling Pathway. Am. J. Hypertens..

[76] Li W., Li W., Leng Y., Xiong Y., Xue R., Chen R., Xia Z. (2020). Mechanism of N-acetylcysteine in alleviating diabetic myocardial ischemia reperfusion injury by regulating PTEN/Akt pathway through promoting DJ-1. Biosci. Rep..

[77] Radomska-Lesniewska D.M., Sadowska A.M., Van Overveld F.J., Demkow U., Zielinski J., De Backer W.A. (2006). Influence of N-acetylcysteine on ICAM-1 expression and IL-8 release from endothelial and epithelial cells. J. Physiol. Pharmacol..

[78] Sadowska A.M., Manuel-y-Keenoy B., Vertongen T., Schippers G., Radomska-Lesniewska D., Heytens E., De Backer W.A. (2006). Effect of N-acetylcysteine on neutrophil activation markers in healthy volunteers: In vivo and in vitro study. Pharmacol. Res..

[79] Radomska-Lesniewska D.M., Skopinska-Rozewska E., Jankowska-Steifer E., Sobiecka M., Sadowska A.M., Hevelke A., Malejczyk J. (2010). N-acetylcysteine inhibits IL-8 and MMP-9 release and ICAM-1 expression by bronchoalveolar cells from interstitial lung disease patients. Pharmacol. Rep..

[80] Fan H., Le J.W., Zhu J.H. (2020). Protective Effect of N-Acetylcysteine Pretreatment on Acute Kidney Injury in Septic Rats. J. Surg. Res..

[81] Liu X., Wang L., Cai J., Liu K., Liu M., Wang H., Zhang H. (2019). N-acetylcysteine alleviates H2O2-induced damage via regulating the redox status of intracellular antioxidants in H9c2 cells. Int. J. Mol. Med..

[82] Yuan C., Wang L., Zhu L., Ran B., Xue X., Wang Z. (2019). N-acetylcysteine alleviated bisphenol A-induced testicular DNA hypermethylation of rare minnow (Gobiocypris rarus) by increasing cysteine contents. Ecotoxicol. Environ. Saf..

[83] Aksit H., Bildik A. (2014). Determination of DNA damage in experimental liver intoxication and role of N-acetyl cysteine. Cell Biochem. Biophys..

[84] Wang L.L., Huang Y.H., Yan C.Y., Wei X.D., Hou J.Q., Pu J.X., Lv J.X. (2016). N-acetylcysteine Ameliorates Prostatitis via miR-141 Regulating Keap1/Nrf2 Signaling. Inflammation.

[85] Yang W., Guo R., Pi A., Ding Q., Hao L., Song Q., Chen L., Dou X., Na L., Li S. (2022). Long non-coding RNA-EN_181 potentially contributes to the protective effects of N-acetylcysteine against non-alcoholic fatty liver disease in mice. Br. J. Nutr..

[86] Kolanu N.D., Syeda Z.R., Joshi N., Singh P., Erukulla M. (2024). The Differential Impact of Medical Therapy and Lifestyle Modification on Cardiovascular Health and Risk of Adverse Cardiovascular Events: A Narrative Review. Cureus.

[87] Straub L.G., Efthymiou V., Grandl G., Balaz M., Challa T.D., Truscello L., Horvath C., Moser C., Rachamin Y., Arnold M. (2019). Antioxidants protect against diabetes by improving glucose homeostasis in mouse models of inducible insulin resistance and obesity. Diabetologia.

[88] Kumar P., Liu C., Hsu J.W., Chacko S., Minard C., Jahoor F., Sekhar R.V. (2021). Glycine and N-acetylcysteine (GlyNAC) supplementation in older adults improves glutathione deficiency, oxidative stress, mitochondrial dysfunction, inflammation, insulin resistance, endothelial dysfunction, genotoxicity, muscle strength, and cognition: Results of a pilot clinical trial. Clin. Transl. Med..

[89] Quesada-Vazquez S., Antolin A., Colom-Pellicer M., Aragones G., Herrero L., Del Bas J.M., Caimari A., Escote X. (2022). Reduction of Obesity and Insulin Resistance through Dual Targeting of VAT and BAT by a Novel Combination of Metabolic Cofactors. Int. J. Mol. Sci..

[90] Garicano Vilar E., Sanz Rojo S., Lopez Oliva S., Martinez S., Terren Lora A., San Mauro Martin I. (2023). Effect of MetioNac(R) in patients with metabolic syndrome who are at risk of metabolic dysfunction associated fatty liver disease: A randomized controlled trial. Nutr. Hosp..

[91] Dludla P.V., Mazibuko-Mbeje S.E., Nyambuya T.M., Mxinwa V., Tiano L., Marcheggiani F., Cirilli I., Louw J., Nkambule B.B. (2019). The beneficial effects of N-acetyl cysteine (NAC) against obesity associated complications: A systematic review of pre-clinical studies. Pharmacol. Res..

[92] Calzadilla P., Gomez-Serrano M., Garcia-Santos E., Schiappacasse A., Abalde Y., Calvo J.C., Peral B., Guerra L.N. (2013). N-Acetylcysteine affects obesity-related protein expression in 3T3-L1 adipocytes. Redox Rep..

[93] Raffaele M., Barbagallo I., Licari M., Carota G., Sferrazzo G., Spampinato M., Sorrenti V., Vanella L. (2018). N-Acetylcysteine (NAC) Ameliorates Lipid-Related Metabolic Dysfunction in Bone Marrow Stromal Cells-Derived Adipocytes. Evid. Based Complement. Altern. Med..

[94] da Silva K.S., Pinto P.R., Fabre N.T., Gomes D.J., Thieme K., Okuda L.S., Iborra R.T., Freitas V.G., Shimizu M.H.M., Teodoro W.R. (2017). N-acetylcysteine Counteracts Adipose Tissue Macrophage Infiltration and Insulin Resistance Elicited by Advanced Glycated Albumin in Healthy Rats. Front. Physiol..

[95] Wolosowicz M., Dajnowicz-Brzezik P., Lukaszuk B., Zebrowska E., Maciejczyk M., Zalewska A., Kasacka I., Chabowski A. (2022). Diverse impact of N-acetylcysteine or alpha-lipoic acid supplementation during high-fat diet regime on fatty acid transporters in visceral and subcutaneous adipose tissue. Adv. Med. Sci..

[96] Manuel A.M., Walla M.D., Dorn M.T., Tanis R.M., Piroli G.G., Frizzell N. (2020). Fumarate and oxidative stress synergize to promote stability of C/EBP homologous protein in the adipocyte. Free Radic. Biol. Med..

[97] Pieri B., Rodrigues M.S., Farias H.R., Silveira G.B., Ribeiro V., Silveira P.C.L., De Souza C.T. (2023). Role of Oxidative Stress on Insulin Resistance in Diet-Induced Obesity Mice. Int. J. Mol. Sci..

[98] Olson D.H., Burrill J.S., Kuzmicic J., Hahn W.S., Park J.M., Kim D.H., Bernlohr D.A. (2017). Down regulation of Peroxiredoxin-3 in 3T3-L1 adipocytes leads to oxidation of Rictor in the mammalian-target of rapamycin complex 2 (mTORC2). Biochem. Biophys. Res. Commun..

[99] Hodun K., Sztolsztener K., Chabowski A. (2021). Antioxidants Supplementation Reduces Ceramide Synthesis Improving the Cardiac Insulin Transduction Pathway in a Rodent Model of Obesity. Nutrients.

[100] Rodrigues M.S., Pieri B., Silveira G.B., Zaccaron R.P., Venturini L.M., Comin V.H., Luiz K.D., Silveira P.C.L. (2020). Reduction of oxidative stress improves insulin signaling in cardiac tissue of obese mice. Einstein.

[101] Sun J., Xu Y., Dai Z., Sun Y. (2009). Intermittent high glucose stimulate MCP-l, IL-18, and PAI-1, but inhibit adiponectin expression and secretion in adipocytes dependent of ROS. Cell Biochem. Biophys..

[102] Fang X., Liu L., Zhou S., Zhu M., Wang B. (2021). N-acetylcysteine inhibits atherosclerosis by correcting glutathione-dependent methylglyoxal elimination and dicarbonyl/oxidative stress in the aorta of diabetic mice. Mol. Med. Rep..

[103] Keshk W.A., Ibrahim M.A., Shalaby S.M., Zalat Z.A., Elseady W.S. (2020). Redox status, inflammation, necroptosis and inflammasome as indispensable contributors to high fat diet (HFD)-induced neurodegeneration; Effect of N-acetylcysteine (NAC). Arch. Biochem. Biophys..

[104] Snodgrass R.G., Boss M., Zezina E., Weigert A., Dehne N., Fleming I., Brune B., Namgaladze D. (2016). Hypoxia Potentiates Palmitate-induced Pro-inflammatory Activation of Primary Human Macrophages. J. Biol. Chem..

[105] Diaz-Saez F., Balcells C., Rossello L., Lopez-Soldado I., Romero M., Sebastian D., Lopez-Soriano F.J., Busquets S., Cascante M., Ricart W. (2024). Neuregulin 4 Downregulation Alters Mitochondrial Morphology and Induces Oxidative Stress in 3T3-L1 Adipocytes. Int. J. Mol. Sci..

[106] Sztolsztener K., Bzdega W., Hodun K., Chabowski A. (2023). N-Acetylcysteine Decreases Myocardial Content of Inflammatory Mediators Preventing the Development of Inflammation State and Oxidative Stress in Rats Subjected to a High-Fat Diet. Int. J. Inflam..

[107] Alnahdi A., John A., Raza H. (2020). Mitigation of Glucolipotoxicity-Induced Apoptosis, Mitochondrial Dysfunction, and Metabolic Stress by N-Acetyl Cysteine in Pancreatic beta-Cells. Biomolecules.

[108] Shin S.K., Cho H.W., Song S.E., Im S.S., Bae J.H., Song D.K. (2020). Oxidative stress resulting from the removal of endogenous catalase induces obesity by promoting hyperplasia and hypertrophy of white adipocytes. Redox Biol..

[109] Chen Y.W., Harris R.A., Hatahet Z., Chou K.M. (2015). Ablation of XP-V gene causes adipose tissue senescence and metabolic abnormalities. Proc. Natl. Acad. Sci. USA.

[110] Sztolsztener K., Michalak D., Chabowski A. (2025). N-acetylcysteine influence on PI3K/Akt/mTOR and sphingolipid pathways in rats with MASLD induced by HFD: A promising new therapeutic purpose. Mol. Cell Endocrinol..

[111] Bauza-Thorbrugge M., Peris E., Zamani S., Micallef P., Paul A., Bartesaghi S., Benrick A., Wernstedt Asterholm I. (2023). NRF2 is essential for adaptative browning of white adipocytes. Redox Biol..

[112] Dai J., Zhang X., Wang Y., Chen H., Chai Y. (2017). ROS-activated NLRP3 inflammasome initiates inflammation in delayed wound healing in diabetic rats. Int. J. Clin. Exp. Pathol..

[113] Soto D., Gomez-Serrano M., Pieralisi A., Calvo J.C., Peral B., Guerra L.N. (2017). N-acetylcysteine inhibits kinase phosphorylation during 3T3-L1 adipocyte differentiation. Redox Rep..

[114] Wang T., Mao X., Li H., Qiao S., Xu A., Wang J., Lei S., Liu Z., Ng K.F., Wong G.T. (2013). N-Acetylcysteine and allopurinol up-regulated the Jak/STAT3 and PI3K/Akt pathways via adiponectin and attenuated myocardial postischemic injury in diabetes. Free Radic. Biol. Med..

[115] Cai J., Huang J., Li D., Zhang X., Shi B., Liu Q., Fang C., Xu S., Zhang Z. (2025). Hippo-YAP/TAZ-ROS signaling axis regulates metaflammation induced by SelenoM deficiency in high-fat diet-derived obesity. J. Adv. Res..

[116] Russell-Guzman J., Americo-Da Silva L., Cadagan C., Maturana M., Palomero J., Estrada M., Barrientos G., Buvinic S., Hidalgo C., Llanos P. (2024). Activation of the ROS/TXNIP/NLRP3 pathway disrupts insulin-dependent glucose uptake in skeletal muscle of insulin-resistant obese mice. Free Radic. Biol. Med..

[117] Argaev-Frenkel L., Rosenzweig T. (2022). Complexity of NAC Action as an Antidiabetic Agent: Opposing Effects of Oxidative and Reductive Stress on Insulin Secretion and Insulin Signaling. Int. J. Mol. Sci..

[118] Schuurman M., Wallace M., Sahi G., Barillaro M., Zhang S., Rahman M., Sawyez C., Borradaile N., Wang R. (2022). N-acetyl-L-cysteine treatment reduces beta-cell oxidative stress and pancreatic stellate cell activity in a high fat diet-induced diabetic mouse model. Front. Endocrinol..

[119] Kaneto H., Kajimoto Y., Miyagawa J., Matsuoka T., Fujitani Y., Umayahara Y., Hanafusa T., Matsuzawa Y., Yamasaki Y., Hori M. (1999). Beneficial effects of antioxidants in diabetes: Possible protection of pancreatic beta-cells against glucose toxicity. Diabetes.

[120] Pereira S., Shah A., George Fantus I., Joseph J.W., Giacca A. (2015). Effect of N-acetyl-l-cysteine on insulin resistance caused by prolonged free fatty acid elevation. J. Endocrinol..

[121] Grzych G., Pekar J.D., Chevalier-Curt M.J., Decoin R., Vergriete P., Henry H., Odou P., Maboudou P., Brousseau T., Vamecq J. (2021). Antioxidants other than vitamin C may be detected by glucose meters: Immediate relevance for patients with disorders targeted by antioxidant therapies. Clin. Biochem..

[122] Ajoolabady A., Pratico D., Bahijri S., Eldakhakhny B., Tuomilehto J., Wu F., Ren J. (2025). Hallmarks and mechanisms of cellular senescence in aging and disease. Cell Death Discov..

[123] Owesny P., Grune T. (2023). The link between obesity and aging—Insights into cardiac energy metabolism. Mech. Ageing Dev..

[124] Salvestrini V., Sell C., Lorenzini A. (2019). Obesity May Accelerate the Aging Process. Front. Endocrinol..

[125] Ou M.Y., Zhang H., Tan P.C., Zhou S.B., Li Q.F. (2022). Adipose tissue aging: Mechanisms and therapeutic implications. Cell Death Dis..

[126] Behtaj D., Ghorbani A., Eslamian G., Malekpour Alamdari N., Abbasi M., Zand H., Shakery A., Shimi G., Sohouli M.H., Fazeli Taherian S. (2024). Ex vivo Anti-Senescence Activity of N-Acetylcysteine in Visceral Adipose Tissue of Obese Volunteers. Obes. Facts.

[127] Sohouli M.H., Eslamian G., Malekpour Alamdari N., Abbasi M., Fazeli Taherian S., Behtaj D., Zand H. (2023). Effects of N-acetylcysteine on aging cell and obesity complications in obese adults: A randomized, double-blind clinical trial. Front. Nutr..

[128] Kumar P., Liu C., Suliburk J., Hsu J.W., Muthupillai R., Jahoor F., Minard C.G., Taffet G.E., Sekhar R.V. (2023). Supplementing Glycine and N-Acetylcysteine (GlyNAC) in Older Adults Improves Glutathione Deficiency, Oxidative Stress, Mitochondrial Dysfunction, Inflammation, Physical Function, and Aging Hallmarks: A Randomized Clinical Trial. J. Gerontol. A Biol. Sci. Med. Sci..

[129] Calzadilla P., Sapochnik D., Cosentino S., Diz V., Dicelio L., Calvo J.C., Guerra L.N. (2011). N-acetylcysteine reduces markers of differentiation in 3T3-L1 adipocytes. Int. J. Mol. Sci..

[130] Pieralisi A., Martini C., Soto D., Vila M.C., Calvo J.C., Guerra L.N. (2016). N-acetylcysteine inhibits lipid accumulation in mouse embryonic adipocytes. Redox Biol..

[131] Li Y., Lee S.H., Piao M., Kim H.S., Lee K.Y. (2023). Metallothionein 3 Inhibits 3T3-L1 Adipocyte Differentiation via Reduction of Reactive Oxygen Species. Antioxidants.

[132] Devlin M.J., Rosen C.J. (2015). The bone-fat interface: Basic and clinical implications of marrow adiposity. Lancet Diabetes Endocrinol..

[133] Ferraro F., Lymperi S., Mendez-Ferrer S., Saez B., Spencer J.A., Yeap B.Y., Masselli E., Graiani G., Prezioso L., Rizzini E.L. (2011). Diabetes impairs hematopoietic stem cell mobilization by altering niche function. Sci. Transl. Med..

[134] Wen W., Li H., Wang C., Chen C., Tang J., Zhou M., Hong X., Cheng Y., Wu Q., Zhang X. (2022). Metabolic dysfunction-associated fatty liver disease and cardiovascular disease: A meta-analysis. Front. Endocrinol..

[135] Ro S.H., Nam M., Jang I., Park H.W., Park H., Semple I.A., Kim M., Kim J.S., Park H., Einat P. (2014). Sestrin2 inhibits uncoupling protein 1 expression through suppressing reactive oxygen species. Proc. Natl. Acad. Sci. USA.

[136] Peris E., Micallef P., Paul A., Palsdottir V., Enejder A., Bauza-Thorbrugge M., Olofsson C.S., Wernstedt Asterholm I. (2019). Antioxidant treatment induces reductive stress associated with mitochondrial dysfunction in adipocytes. J. Biol. Chem..

[137] Garcia-Serrano A.M., Vieira J.P.P., Fleischhart V., Duarte J.M.N. (2023). Taurine and N-acetylcysteine treatments prevent memory impairment and metabolite profile alterations in the hippocampus of high-fat diet-fed female mice. Nutr. Neurosci..

[138] Hurley M.M., Resch J.M., Maunze B., Frenkel M.M., Baker D.A., Choi S. (2016). N-acetylcysteine decreases binge eating in a rodent model. Int. J. Obes..

[139] Sketriene D., Battista D., Perry C.J., Sumithran P., Lawrence A.J., Brown R.M. (2021). N-acetylcysteine reduces addiction-like behaviour towards high-fat high-sugar food in diet-induced obese rats. Eur. J. Neurosci..

[140] John P., Marenco R.E.F. (2018). The Clinical Use of N-Acetylcysteine in Cardiology.

[141] Bartkowiak K., Bartkowiak M., Jankowska-Steifer E., Ratajska A., Kujawa M., Aniolek O., Niderla-Bielinska J. (2024). Metabolic Syndrome and Cardiac Vessel Remodeling Associated with Vessel Rarefaction: A Possible Underlying Mechanism May Result from a Poor Angiogenic Response to Altered VEGF Signaling Pathways. J. Vasc. Res..

[142] Xu S., Ilyas I., Little P.J., Li H., Kamato D., Zheng X., Luo S., Li Z., Liu P., Han J. (2021). Endothelial Dysfunction in Atherosclerotic Cardiovascular Diseases and Beyond: From Mechanism to Pharmacotherapies. Pharmacol. Rev..

[143] Mushtaq I., Bashir Z., Sarwar M., Arshad M., Ishtiaq A., Khan W., Khan U., Tabassum S., Ali T., Fatima T. (2021). N-Acetyl Cysteine, Selenium, and Ascorbic Acid Rescue Diabetic Cardiac Hypertrophy via Mitochondrial-Associated Redox Regulators. Molecules.

[144] Marian A.J., Tan Y., Li L., Chang J., Syrris P., Hessabi M., Rahbar M.H., Willerson J.T., Cheong B.Y., Liu C.Y. (2018). Hypertrophy Regression With N-Acetylcysteine in Hypertrophic Cardiomyopathy (HALT-HCM): A Randomized, Placebo-Controlled, Double-Blind Pilot Study. Circ. Res..

[145] Kelley R.C., Lapierre S.S., Muscato D.R., Hahn D., Christou D.D., Ferreira L.F. (2022). Cardiac and respiratory muscle responses to dietary N-acetylcysteine in rats consuming a high-saturated fat, high-sucrose diet. Exp. Physiol..

[146] Sivasinprasasn S., Chattipakorn K., Pratchayasakul W., Chattipakorn S.C., Chattipakorn N. (2025). N-Acetylcysteine enhances low-dose estrogen efficacy against ischemia-reperfusion injury in estrogen-deprived obese insulin-resistant rats. Menopause.

[147] Voghel G., Thorin-Trescases N., Farhat N., Mamarbachi A.M., Villeneuve L., Fortier A., Perrault L.P., Carrier M., Thorin E. (2008). Chronic treatment with N-acetyl-cystein delays cellular senescence in endothelial cells isolated from a subgroup of atherosclerotic patients. Mech. Ageing Dev..

[148] Ulloque-Badaracco J.R., Hernandez-Bustamante E.A., Alarcon-Braga E.A., Al-Kassab-Cordova A., Cabrera-Guzman J.C., Herrera-Anazco P., Benites-Zapata V.A. (2023). Vitamin B12, folate, and homocysteine in metabolic syndrome: A systematic review and meta-analysis. Front. Endocrinol..

[149] Ventura P., Panini R., Pasini M.C., Scarpetta G., Salvioli G. (1999). N -Acetyl-cysteine reduces homocysteine plasma levels after single intravenous administration by increasing thiols urinary excretion. Pharmacol. Res..

[150] Wiklund O., Fager G., Andersson A., Lundstam U., Masson P., Hultberg B. (1996). N-acetylcysteine treatment lowers plasma homocysteine but not serum lipoprotein(a) levels. Atherosclerosis.

[151] Hildebrandt W., Sauer R., Bonaterra G., Dugi K.A., Edler L., Kinscherf R. (2015). Oral N-acetylcysteine reduces plasma homocysteine concentrations regardless of lipid or smoking status. Am. J. Clin. Nutr..

[152] Faghfouri A.H., Zarezadeh M., Tavakoli-Rouzbehani O.M., Radkhah N., Faghfuri E., Kord-Varkaneh H., Tan S.C., Ostadrahimi A. (2020). The effects of N-acetylcysteine on inflammatory and oxidative stress biomarkers: A systematic review and meta-analysis of controlled clinical trials. Eur. J. Pharmacol..

[153] Ardissino D., Merlini P.A., Savonitto S., Demicheli G., Zanini P., Bertocchi F., Falcone C., Ghio S., Marinoni G., Montemartini C. (1997). Effect of transdermal nitroglycerin or N-acetylcysteine, or both, in the long-term treatment of unstable angina pectoris. J. Am. Coll. Cardiol..

[154] Martina V., Masha A., Gigliardi V.R., Brocato L., Manzato E., Berchio A., Massarenti P., Settanni F., Della Casa L., Bergamini S. (2008). Long-term N-acetylcysteine and L-arginine administration reduces endothelial activation and systolic blood pressure in hypertensive patients with type 2 diabetes. Diabetes Care.

[155] Barrios V., Calderon A., Navarro-Cid J., Lahera V., Ruilope L.M. (2002). N-acetylcysteine potentiates the antihypertensive effect of ACE inhibitors in hypertensive patients. Blood Press..

[156] Marchetti G., Lodola E., Licciardello L., Colombo A. (1999). Use of N-acetylcysteine in the management of coronary artery diseases. Cardiologia.

[157] Sun M., Lu Z., Chen W.M., Lv S., Fu N., Yang Y., Wang Y., Miao M., Wu S.Y., Zhang J. (2025). N-acetylcysteine therapy reduces major adverse cardiovascular events in patients with type 2 diabetes mellitus. Atherosclerosis.

[158] Talasaz A.H., Khalili H., Fahimi F., Jenab Y., Broumand M.A., Salarifar M., Darabi F. (2014). Effects of N-acetylcysteine on the cardiac remodeling biomarkers and major adverse events following acute myocardial infarction: A randomized clinical trial. Am. J. Cardiovasc. Drugs.

[159] Yesilbursa D., Serdar A., Senturk T., Serdar Z., Sag S., Cordan J. (2006). Effect of N-acetylcysteine on oxidative stress and ventricular function in patients with myocardial infarction. Heart Vessel..

[160] Arstall M.A., Yang J., Stafford I., Betts W.H., Horowitz J.D. (1995). N-acetylcysteine in combination with nitroglycerin and streptokinase for the treatment of evolving acute myocardial infarction. Safety and biochemical effects. Circulation.

[161] Sajkowska A., Wykretowicz A., Szczepanik A., Kempa M., Minczykowski A., Wysocki H. (1999). Fibrinolytic therapy and n-acetylocysteine in the treatment of patients with acute myocardial infarction: Its influence on authentic plasma hydroperoxide levels and polymorphonuclear neutrophil oxygen metabolism. Cardiology.

[162] Thiele H., Hildebrand L., Schirdewahn C., Eitel I., Adams V., Fuernau G., Erbs S., Linke A., Diederich K.W., Nowak M. (2010). Impact of high-dose N-acetylcysteine versus placebo on contrast-induced nephropathy and myocardial reperfusion injury in unselected patients with ST-segment elevation myocardial infarction undergoing primary percutaneous coronary intervention. The LIPSIA-N-ACC (Prospective, Single-Blind, Placebo-Controlled, Randomized Leipzig Immediate PercutaneouS Coronary Intervention Acute Myocardial Infarction N-ACC) Trial. J. Am. Coll. Cardiol..

[163] Pasupathy S., Tavella R., Grover S., Raman B., Procter N.E.K., Du Y.T., Mahadavan G., Stafford I., Heresztyn T., Holmes A. (2017). Early Use of N-acetylcysteine With Nitrate Therapy in Patients Undergoing Primary Percutaneous Coronary Intervention for ST-Segment-Elevation Myocardial Infarction Reduces Myocardial Infarct Size (the NACIAM Trial [N-acetylcysteine in Acute Myocardial Infarction]). Circulation.

